# Microbial Transformation of Flavonoids by *Isaria fumosorosea* ACCC 37814

**DOI:** 10.3390/molecules24061028

**Published:** 2019-03-15

**Authors:** Fangmin Dou, Zhi Wang, Guiying Li, Baoqing Dun

**Affiliations:** The National Key Facility for Crop Gene Resources and Genetic Improvement, Institute of Crop Sciences, Chinese Academy of Agricultural Sciences, 12 Zhongguancun South Street, Beijing 100081, China; doufangmin@hotmail.com (F.D.); wangzhi@caas.cn (Z.W.); liguiying@caas.cn (G.L.)

**Keywords:** *Isaria fumosorosea*, flavonoids, microbial transformation, methylglycosylation

## Abstract

Glycosylation is an efficient strategy to modulate the solubility, stability, bioavailability and bioactivity of drug-like natural products. Biological methods, such as whole-cell biocatalyst, promise a simple but highly effective approach to glycosylate biologically active small molecules with remarkable regio- and stereo-selectivity. Herein, we use the entomopathogenic filamentous fungus *Isaria fumosorosea* ACCC 37814 to biotransform a panel of phenolic natural products, including flavonoids and anthraquinone, into their glycosides. Six new flavonoid (4-*O*-methyl)glucopyranosides are obtained and structurally characterized using high resolution mass and nuclear magnetic resonance spectroscopic techniques. These compounds further expand the structural diversity of flavonoid glycosides and may be used in biological study.

## 1. Introduction

**Figure 1:**
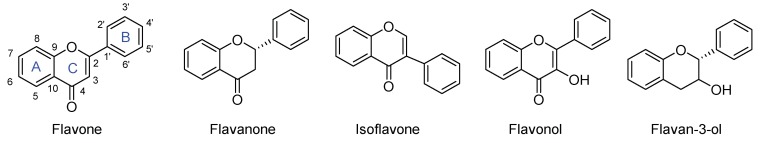
Basic structures of major flavonoid subclasses.

Flavonoids constitute the largest group of plant-derived polyphenol secondary metabolites which could be found in various vegetables, fruits, nuts, grains, spices, wine and tea. The daily intake of flavonoids can range from 50 to 800 mg, depending on the consumption of flavonoid-containing foods and beverages [1]. Many studies have shown that flavonoids not only have beneficial effects to human health, such as antioxidative, cardioprotective and neuroprotective properties but also display a wide range of pharmacological activities including anticancer, anti-inflammatory, antibiotic and so forth [2,3,4]. The basic flavonoid structure consists of 15 carbon atoms that arranged in a C6-C3-C6 triple-ring system in which two aromatic rings (ring A and B) are connected by a C3 moiety (ring C). Based on the oxidation level and substitution pattern of ring C, flavonoids can be categorized into several subclasses such as flavones, flavanones, isoflavones, flavonols, flavanonols and flavan-3-ols (Figure 1). The structural diversity of individual flavonoids within these subclasses, however, arises from the variations of substitution pattern on ring A and B [1]. Most naturally occurring flavonoids exist in the form of glycosides in which single or multiple sugar moieties are attached to the flavonoid scaffold via *O*- or *C*-glycosidic bonds [2]. Many clinically important natural products, such as the antibiotic vancomycin and erythromycin and the antineoplastic doxorubicin, require the sugar moiety to maintain their bioactivities [5,6,7]. However, glycosylation of flavonoids has a much more complicated impact on their biological activities which is not only influenced by the structural variations of the flavonoid core and the sugar unit but also by the number of sugar moieties and the position that the sugars are attached to [2,8,9]. Despite the fact that flavonoid aglycones usually exhibit stronger activities than their sugar-conjugates in cell-based bioassays, the potential application of aglycones are often limited by their insufficient solubility in organic and aqueous solvents, low bioavailability after oral administration and rapid metabolism in vivo [10,11]. Flavonoid glycosides, on the other hand, have satisfying solubility and in many cases display improved bioavailability when comparing to the aglycones. For instance, quercetin 3- and 4′-*O*-β-d-glucosides are much more efficiently absorbed than quercetin and quercetin 3-*O*-β-d-rutinoside in the small intestine [10,12], which probably due to the active transport of glucosides by Na-dependent glucose transporter 1 (SGLT1) presented in the brush-border membrane of the small intestine [13]. As glycosylation plays such a substantial role in modulating the physicochemical, biological and metabolic properties of flavonoids, it is important to further expand the structural diversity of flavonoid glycosides via chemical and/or biological approaches.

Chemical glycosylation of structurally complex natural products often requires laborious protection-deprotection procedures to achieve regio- and stereo-selectivity. By contrast, biological methods, such as whole-cell biocatalysis, in vivo total biosynthesis and in vitro enzymatic reaction using isolated glycosyltransferases, offer alternative approaches to realize one-pot and one-step regio- and stereospecific glycosylations that proceed under mild conditions and avoid the use of toxic reagents [9,14]. Filamentous fungi, especially species of genera *Aspergillus* and *Beauveria*, are rich sources of whole-cell biocatalyst for the glycosylation of natural products [15,16,17]. Entomopathogenic fungi are an ecologically highly specialized group of microorganisms that infect, kill or seriously disable insects and many other arthropods. *Isaria fumosorosea* is an important member of this group of fungi which is isolated from over 40 species of arthropods and also presents in soil, water and plants of every continent except for Antarctica [18]. *I. fumosorosea* alone or in combination with other entomopathogenic fungal species have been successfully commercialized as mycopesticides in America, Europe and Asia [11]. Species of genus *Isaria* produce a wide range of biologically active natural products, such as the immunosuppressant sphingoid ISP-I [19], the antioxidative pseudo-dipeptide hanasanagin [20], the insecticidal cyclodepsipeptides, isariins, isarolides and safelins/isaridins [21,22,23,24], the carotane-type sesquiterpenes, trichocaranes E and F, CAF-603 and trichocarane C [25] and the novel skeleton polyketide tenuipyrone [26]. More importantly to us, several *I. fumosorosea* species have shown prominent ability in biotransforming a variety of natural products into their glycosides [11,27,28,29].

To further exploit the biocatalytic potential of *I. fumosorosea* and to expand the chemical diversity of flavonoid glycosides, we used *Isaria fumosorosea* ACCC 37814 as whole-cell biocatalyst to glycosylate eight flavonoids which cover the major subclasses of flavonoid, such as flavanone (naringenin **1** and hesperetin **6**), flavone (luteolin **2**, diosmetin **3** and apigenin **7**), isoflavone (formononetin **4** and genistein **8**) and flavonol (kaempferol **5**), as well as another two phenolic compounds (emodin **9** and curcumin **10**). As a result, six new flavonoid methylglucopyranosides, namely 5,7-dihydroxyflavanone 4′-*O*-β-d-(4-*O*-methyl)glucopyranoside **1b**, 5-hydroxyflavanone 4′,7-di-*O*-β-d-(4-*O*-methyl)glucopyranoside **1d**, 4′,5,7-trihydroxyflavone 3′-*O*-β-d-(4-*O*-methyl)glucopyranoside **2a**, 3′,5,7-trihydroxyflavone 4′-*O*-β-d-(4-*O*-methyl)glucopyranoside **2b**, 5,7-dihydroxy-4′-methoxyflavone 3′-*O*-β-d-(4-*O*-methyl)glucopyranoside **3a** and 4′-methoxyisoflavone 7-*O*-β-d-(4-*O*-methyl)glucopyranoside **4a**, along with two known compounds 4′,5-dihydroxyflavanone 7-*O*-β-d-(4-*O*-methyl)glucopyranoside **1a** and 5,7-dihydroxyflavanone 4′-*O*-β-d-glucopyranoside **1c** were obtained and structurally characterized.

## 2. Results and Discussion

### 2.1. Biotransformation Assay

**Figure 2:**
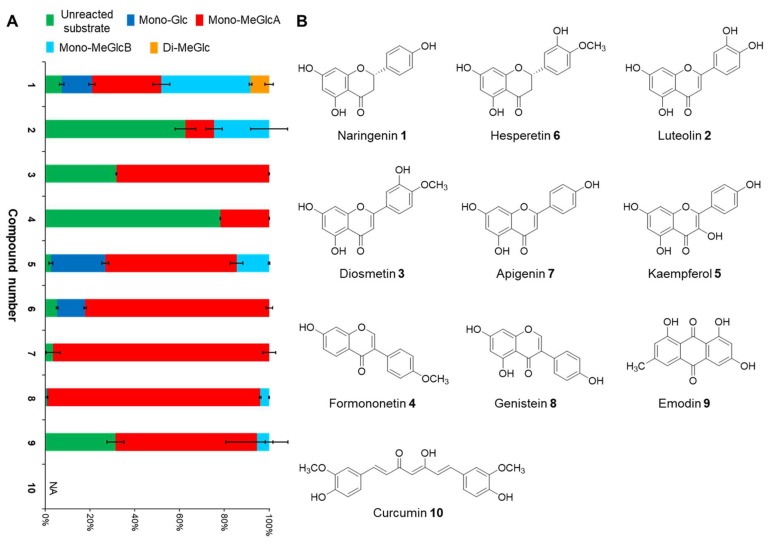
The biotransformation of phenolic substrates by *I. fumosorosea* ACCC 37814. (**A**) Total conversion rate [%] of each tested substrate. All data represent the means ± SDs in three independent experiments. Mono-Glc: mono-glucoside; Mono-MeGlcA and –B: mono-(4-*O*-methyl)glucoside regioisomers; Di-MeGlc: di-(4-*O*-methyl)glucoside. NA: not applicable (**B**) Chemical structures of substrates **1**–**10**. See Supplementary Materials, Table S1 for tabulated data.

Small-scale fermentations of *I. fumosorosea* ACCC 37814 in the presence of each substrate were carried out to screen for all possible glycosylated products. The ethyl acetate extract of each fermentation culture was analyzed by HPLC-HRMS/MS and the potential glycosylated products were identified after examining their LC and HRMS/MS spectra. Satisfactorily, all tested compounds except for curcumin **10** were converted by *I. fumosorosea* ACCC 37814 into at least one glycosylated products. The LC peak areas (recorded at 300 nm) of all products and the unreacted substrate were used to calculate the substrate-to-product conversion rate for each compound (Figure 2 and Supplementary Materials, Table S1). In all cases, mono-methylglucoside was produced as the major product, while mono-glucoside was also detected for some flavonoids such as naringenin **1**, kaempferol **5** and hesperetin **6**. Mono-methylglucoside regioisomers were presented in extracts of naringenin **1**, luteolin **2**, kaempferol **5**, genistein **8** and emodin **9** but only naringenin had di-methylglucosylated product (Figure 2 and Supplementary Materials, Table S1). The precise position to which the glycosyl and methyl substituents were attached, however, remained unclear at this point where NMR measurements were required to unambiguously characterize the structure.

### 2.2. Structure Characterization

**Figure 3:**
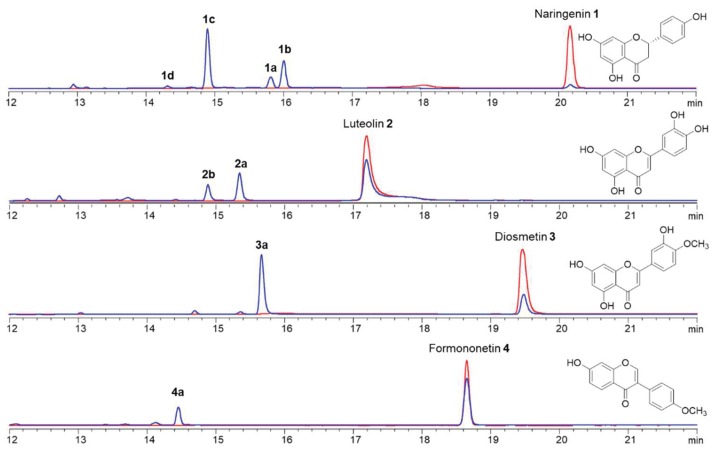
Product profiles of flavonoids **1**–**4** biotransformed in the *I. fumosorosea* ACCC 37814 cultures (reverse phase High Performance Liquid Chromatography (HPLC) traces recorded at 300 nm). HPLC traces of each standard substrate (colored in red) were used as references.

Scale-up biotransformations of flavonoids **1**, **2**, **3** and **4** by *I. fumosorosea* ACCC 37814 were carried out to obtain enough amount of their glycosides for NMR measurements. The fermentation culture was extracted by ethyl acetate and the resulting crude extract was subjected to silica gel column chromatography and semi-preparative HPLC to give the single compounds **1a**–**1d**, **2a**, **2b**, **3a** and **4a** (Figure 3). Their structures were unambiguously characterized by mass and NMR spectroscopic techniques.

**Scheme 1:**
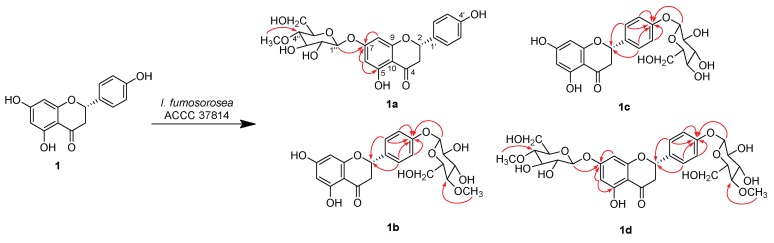
The microbial transformation of naringenin **1** in the *I. fumosorosea* ACCC 37814 culture. Key Heteronuclear Multiple Bond Correlations (HMBC) correlations of each product are indicated by red arrows.

2.3 mg of **1a**, 3.2 mg of **1b**, 6.5 mg of **1c** and 1.5 mg of **1d** were isolated from the scale-up fermentation culture of *I. fumosorosea* ACCC 37814 (Scheme 1). The negative mode HRESIMS spectra of **1a** and **1b** displayed [M − H]^−^ ion peaks at *m/z* 447.1289 and 447.1294, respectively, which were 176 amu larger than that of naringenin **1** and thus indicated the presence of a methylated hexose unit in their structures. This was further supported by ^1^H- and ^13^C-NMR spectra of **1a** and **1b**, which were quite similar, and both showed characteristic resonances of a naringenin core and a methylated d-glucosyl substituent (Table 1). Next, comprehensive analysis of HSQC and HMBC spectra revealed that **1a** and **1b** were regioisomers in which the sugar substituent was attached to different positions. The HMBC spectrum of **1a** displayed correlations of the anomeric proton (δ_H_ 5.01, d, *J* = 7.8 Hz) and two aromatic protons (H-6, δ_H_ 6.15, brs; H-8, δ_H_ 6.13, brs) with the oxygen-bearing aromatic carbon at δ_C_ 165.1 (C-7), revealing that the d-glucosyl substituent was connected to OH-7 of naringenin (Scheme 1). Moreover, the large coupling constant of the anomeric proton confirmed the β-configuration of the glucosidic bond. Similarly, the d-glucose fragment in **1b** was confirmed to be connected to C-4′ of naringenin via an *O*-β-d-glycosidic bond because of the large coupling constant of the anomeric proton (δ_H_ 4.91, d, *J* = 7.7 Hz) and HMBC interaction between the anomeric proton and the oxygen-bearing aromatic carbon at δ_C_ 157.4 (C-4′). In both cases, HMBC correlations between the methoxy protons (**1a**: δ_H_ 3.44, s; **1b**: δ_H_ 3.45, s) and C-4 of the glucose moiety (**1a**: δ_C_ 78.8; 1a: δ_C_ 79.0) confirmed that the methylation has been taken place on the OH-4 of the glucose fragment (Scheme 1). Hence, the structures of **1a** and **1b** were defined as 4′,5-dihydroxyflavanone 7-*O*-β-d-(4-*O*-methyl)glucopyranoside and 5,7-dihydroxyflavanone 4′-*O*-β-d-(4-*O*-methyl)glucopyranoside, respectively.

The HRESIMS-determined molecular weight of **1c** was 14 amu lower than those of **1a** and **1b**. Moreover, ^1^H- and ^13^C-NMR spectra of **1c** were almost superimposable to those of **1b**, except for the absence of the methoxy signals and some chemical shift variations on C-3, C-4 and C-5 of the glucosyl substituent (Table 1). These evidence indicated that **1c** has a glucosyl substituent, instead of a 4-*O*-methylated glucosyl group, attached to the 4′-OH of naringenin. Comprehensive analysis of HSQC and HMBC spectra of **1c** further supported this speculation (Scheme 1) and thus the structure of **1c** was elucidated as 5,7-dihydroxyflavanone 4′-*O*-β-d-glucopyranoside.

The HRESIMS determined molecular weight of **1d** was 176 amu larger than those of **1a** and **1b** and its ^1^H- and ^13^C-NMR spectra showed resonance signals corresponding to two methylated-glucosyl substituents (Table 1), suggesting that **1d** was the di-(4-*O*-methyl)glucopyranosyl substituted derivative of naringenin. After careful examination of the 2D-NMR spectra (Scheme 1), the structure of **1d** was defined as 5-hydroxyflavanone 4′,7-di-*O*-β-d-(4-*O*-methyl)glucopyranoside.

**Scheme 2:**
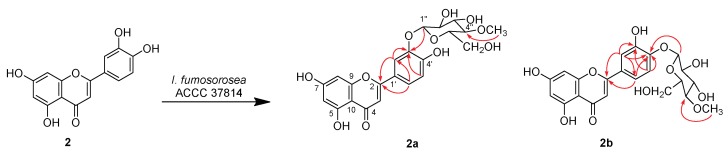
The microbial transformation of luteolin **2** in the *I. fumosorosea* ACCC 37814 culture. Key HMBC correlations of each product are indicated by red arrows.

The scale-up biotransformation of luteolin **2** in the culture of *I. fumosorosea* ACCC 37814 resulted in 2.8 mg of product **2a** and 2.3 mg of product **2b** (Scheme 2). The (−)-HRESIMS spectra of **2a** and **2b** displayed [M − H]^−^ ions at *m/z* 461.1100 and 461.1097, respectively, which were consistent with that of (4-*O*-methyl)glucosyl substituted luteolin. ^1^H- and ^13^C-NMR spectra of **2a** and **2b** showed resonance signals corresponding to a luteolin core structure, a d-glucose unit and a methoxy functionality. Apparently, **2a** and **2b** were regio-isomers which have the sugar fragment attached to different positions of luteolin. In the case of **2a**, the coupling constant of the anomeric proton (δ_H_ 4.93, d, *J* = 7.8 Hz) along with the HMBC correlation between the anomeric proton and the oxygen-bearing aromatic carbon at δ_C_ 145.7 (C-3′) confirmed that the glucosyl substituent was connected to C-3′ of luteolin via a *O*-β-glucosidic linkage (Scheme 2). By contrast, the HMBC spectrum of **2b** exhibited cross peak between the anomeric proton (δ_H_ 4.92, d, *J* = 7.8 Hz) and the oxygen-bearing aromatic carbon at δ_C_ 148.4 (C-4′), confirming an *O*-β-glucosidic linkage between the C-4′ of luteolin and the anomeric carbon of the sugar unit (Scheme 2). Based on HMBC correlation between the methoxy protons (**2a** and **2b**: δ_H_ 3.47, s) and C-4 of the glucose moiety (**2a**: δ_C_ 79.3; **2b**: δ_C_ 79.0), the methoxy groups in both **2a** and **2b** were confirmed to be attached to C-4 of the glucosyl substituent. Hence, the structures of **2a** and **2b** were established as 4′,5,7-trihydroxyflavone 3′-*O*-β-d-(4-*O*-methyl)glucopyranoside and 3′,5,7-trihydroxyflavone 4′-*O*-β-d-(4-*O*-methyl)glucopyranoside, respectively.

**Scheme 3:**
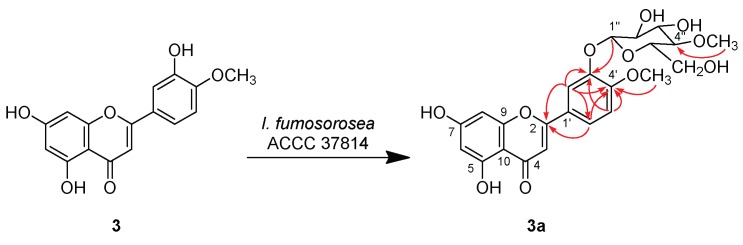
The microbial transformation of diosmetin **3** in the *I. fumosorosea* ACCC 37814 culture. Key HMBC correlations of each product are indicated by red arrows.

2.0 mg of compound **3a** was isolated from the scale-up fermentation culture of *I. fumosorosea* ACCC 37814. The HRESIMS determined molecular weight of **3a** was consistent with that of (4-*O*-methyl)glucosylated derivative of diosmetin **3**. Detailed analysis of 1D- and 2D-NMR spectra further confirmed that **3a** is the methylglucosylated derivative of diosmetin. HMBC interaction between the anomeric proton (δ_H_ 5.17, d, *J* = 7.8 Hz) and the oxygen-bearing aromatic carton at δ_C_ 146.5 (C-3′) indicated that the glucose unit was attached to OH-3′ of diosmetin (Scheme 3). The β-configuration of the glucosidic bond was proved by the coupling constant of the anomeric proton. HMBC correlation between the methoxy protons (δ_H_ 3.47, s) and the oxygen-bearing sp^3^-carbon at δ_C_ 79.1 (C-4′′) confirmed that the methylation has been taken place on the 4-OH of the glucose moiety. Thus, the structure of **3a** was defined as 5,7-dihydroxy-4′-methoxyflavone 3′-*O*-β-d-(4-*O*-methyl)glucopyranoside.

**Scheme 4:**
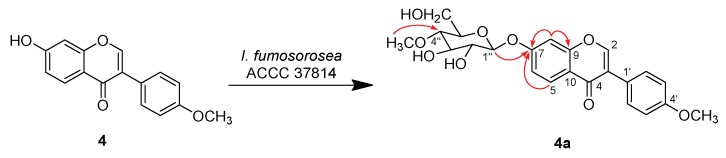
The microbial transformation of formononetin **3** in the *I. fumosorosea* ACCC 37814 culture. Key HMBC correlations of each product are indicated by red arrows.

2.0 mg of compound **4a** was purified from the scale-up biotransformation of formononetin **4** in the culture of *I. fumosorosea* ACCC 37814. The negative mode HRESIMS spectrum of **4a** displayed a [M − H]^−^ ion at *m*/*z* 489.1422, indicating **4a** was the methylglucosylated derivative of formononetin. This speculation was supported by the presence of resonance signals corresponding to a formononetin core, a d-glucose unit and a methoxy functional group in the ^1^H- and ^13^C-NMR spectra of **4a**. HMBC interaction between the anomeric proton (δ_H_ 5.14, d, *J* = 7.8 Hz ) and the oxygen-bearing aromatic carton at δ_C_ 161.3 (C-7) as well as the large coupling constant of this anomeric proton indicated that the glucose fragment was attached to C-7 of formononetin via an *O*-β-glucosidic linkage. The HMBC spectrum of **4a** also displayed cross peak between the methoxy protons (δ_H_ 3.47, s) and the oxygen-bearing sp^3^-carbon at δ_C_ 78.9 (C-4′′), confirming that the C-4 of the glucose moiety has a methoxy substituent (Scheme 4). Hence, the structure of **4a** was elucidated as 4′-methoxyisoflavone 7-*O*-β-d-(4-*O*-methyl)glucopyranoside.

Most of the naturally occurring flavonoids exist in the form of *O*-glycosides and flavone and flavonol *O*-glycosides account for the largest two groups of flavonoid glycosides [2]. Theoretically speaking, any free phenolic hydroxyl group on the flavonoid scaffold can be glycosylated except for OH-5 which forms hydrogen bond with the C-4 carbonyl group and thus has relatively low reactivity [8]. However, nature seems to favor some positions rather than others. The majority of naturally occurring flavones, flavanones and isoflavones tend to be glycosylated at OH-7, while flavonols and flavanols usually have sugar fragments attached to OH-3 and/or OH-7 [2]. Therefore, it is interesting that *I. fumosorosea* ACCC 37814 prefers to glycosylate the OH-3′ or OH-4′ on ring B of flavones such as luteolin and diosmetin. By contrast, the widely studied biocatalyst, *B. bassiana*, which is also an entomopathogenic filamentous fungus and genetically close to *I. fumosorosea*, favors OH-7 as methylglucosylation site [14]. 

The biosynthesis of natural flavonoid *O*-glycosides in plant cells are catalyzed by various uridine diphosphate-dependent glycosyltransferases (UGTs). Over the past two decades, highly efficient biosynthesis of flavonoid *O*-glycosides has been achieved by using genetically engineered *Escherichia coli* strains that harbor UGTs from *Arabidopsis thaliana*, *Oryza sativa*, *Medicago truncatula* and so forth [30,31,32]. Bacteria also utilize a wide range of UGTs to glycosylate their secondary metabolites. *E. coli* strains that expressing heterologous bacterial UGTs were used to biosynthesize not only natural flavonoid *O*-glucosides [33,34,35] but also “unnatural” flavonoid glycosides which incorporate amino- and deoxysugars in their structures [36,37]. Despite the fact that fungi have long been used as whole-cell biocatalyst for the glycosylation of a variety of natural products, only a few fungal UGTs have been identified and functional characterized so far [9,14,38]. Very recently, Xie et al. identified a glycotransferase-methyltransferase (GT-MT) biosynthetic module from *B. bassiana* which proficiently synthesized (4-*O*-methyl)glucopyranosides of various phenolic substances, including benzendiol lactones, anthraquinones and flavonoids, in *Saccharomyces cerevisiae* host [14]. Recent studies and our present work demonstrated that *I. fumosorosea* is capable of methylglucosylating a number of flavonoid scaffolds and prefers the free aromatic hydroxyl group on ring B as glycosylation site [11,27,28,29]. These results indicate that *I. fumosorosea* is a promising source to exploit novel fungal GT-MT module that exhibit different chemo- and regio-selectivity than those from *B. bassiana*.

Many studies have proved that glycosylation significantly improves the aqueous solubility of natural products such as flavonoids, while methylation on one of the hydroxyl groups of the sugar moiety does not significantly alter the solubility [5,14]. However, the biological and pharmacological significance of the 4-*O*-methylation of the sugar moiety is less clear. Flavonoids and their glycosides undergo intense metabolism by both intestine epithelium and gut microbiota after they are taken into human body, which eventually diminishes the beneficial effects of flavonoids observed in vitro [12]. A recent study has shown that attaching methyl group to the OH-4 of glucose moiety can substantially avert the rapid de-glucosylation process of benzendiol lactone glucosides in both yeast and mammalian cell models [14] and thus may increase the in vivo stability and bioavailability of these drug-like compounds. Consequently, the effect of methylglucosylation of flavonoids would be an interesting topic to further explore.

## 3. Conclusions

The current study describes the microbial transformation of natural phenolics using the entomopathogenic filamentous fungus *I. fumosorosea* ACCC 37814. Nine out of ten substrates were converted to their glycosides in the culture of *I. fumosorosea* ACCC 37814 after four days of biotransformation. Mono-methylglucosides are the major products for all substrates but di- methylglucosylated and mono-glucosylated products were also detected in several cases. Four substrates representing different sub-classes of flavonoid were selected for scale-up fermentation. As a result, six new flavonoid glycosides and two known compounds were isolated from the scale-up cultures and their structures were unambiguously characterized. The results indicated that *I. fumosorosea* ACCC 37814 prefers free aromatic hydroxyl groups on ring B of flavonoid scaffold as glycosylation site and could be developed as regio-selective biocatalyst for the methylglucosylation of phenolic natural products.

## 4. Materials and Methods

### 4.1. General Experimental Orocedures

All substrates were purchased from Shanghai Yuanye Bio-Technology Co., Ltd. (Shanghai, China). Samples were routinely analyzed on an Agilent 1260 Infinity II HPLC instrument (Santa Clara, CA, USA). The HPLC was equipped with an Agilent Eclipse Plus C_18_ RRHD column (1.8 µm, 2.1 mm × 50 mm) and eluted with a linear gradient of 10–95% of acetonitrile-water for 20 min, 95% acetonitrile-water for 10 min and drop down to 10% in 5 min, then 10% acetonitrile-water for 5 min at a flow rate of 0.5 mL/min. ^1^H-, ^13^C-, HSQC- and HMBC-NMR spectra were recorded on an Agilent 600 DD2 spectrometer. Chemical shift values (δ) are given in parts per million (ppm) and the coupling constants (*J* values) are in Hz. Chemical shifts were referenced to the residual solvent peaks of DMSO-*d*_6_. HPLC-HRESIMS spectra were acquired on an Agilent 1290 Infinity II HPLC coupled with an Agilent QTOF 6530 instrument operated in negative ion mode using capillary and cone voltages of 3.6 kV and 40–150 V, respectively. For accurate mass measurements the instrument was calibrated each time using a standard calibration mix (Agilent) in the range of *m*/*z* 150–1900. 

### 4.2. Culture and Biotransformation Procedures

The fungus *Isaria fumosorosea* ACCC 37814, acquired from the Agricultural Culture Collection of China (ACCC), was maintained on potato-dextrose agar (PDA, Difco, Sparks, MD, USA) at 26 °C for 7 days. Spores were collected after 10 days of growth by flooding the plates with sterile water (0.1% Triton-X100). For small scale biotransformation experiment, the collected spores with 10^6^/mL concentration were inoculated into a 100 mL Erlenmeyer flask containing 50 mL potato-dextrose broth (PDB, Difco, Sparks, MD, USA) medium. The flask was placed in a rotary shaker at 25 °C and shook at 220 rpm for three days. After that, 0.5 mg of substrates (10 uL DMSO solution of substrate at the concentration of 50 mg/mL) was added and the flask was maintained under the same conditions for an additional four days. Two controls were incubated under the same conditions: (1) *I. fumosorosea* ACCC 37814 fermentation broth with the addition of substrate-free DMSO solution and (2) microorganism-free PDB medium with the addition of DMSO solution containing the substrate. For large scale fermentation, a total of 3 L fermentation culture and 75 mg of substrates were used (2.5 mg/flask) in the 250 mL flasks, each containing 100 mL of PDB and incubated under the same conditions as described for the small scale experiment. 

### 4.3. Extraction, Isolation and Purification of the Products

The scale-up fermentation cultures were combined and extracted with an equal volume of ethyl acetate for three times. The extracts were pooled and dried in vacuo to give the crude extracts Ⅰ (481.6 mg/L), Ⅱ (844.0 mg/L), Ⅲ (868.1 mg/L) and Ⅳ (868.1 mg/L) for substrates **1**–**4**, respectively. Each crude extract was first subjected to silica gel column chromatography and eluted with a gradient of dichloromethane/methanol (100:0, 98:2, 95:5, 93:7 and 90:10, *v*/*v*) to give five fractions (Fr. A–E). These fractions were analyzed by LC-MS instrument (Agilent Technologies, Santa Clara, CA, USA) to detect potential products. Fractions containing the target compounds were subsequently purified by semi-preparative HPLC on an Agilent Eclipse XDB-C18 reversed-phase column (5 μm, 9.4 mm × 250 mm) using an Agilent 1260 Infinity II instrument. Specifically, Fr. D of extract I was isolated using acetonitrile-water (25:75, *v*/*v*) as eluent at the flow rate of 2 mL/min to give compound **1a** (2.3 mg, *t_R_* = 23.2 min), compound **1b** (3.2 mg, *t*_R_ = 27.3 min), compound **1c** (6.5 mg, *t*_R_ = 18.5 min) and compound **1d** (1.5 mg, *t*_R_ = 13.4 min); Fr. D of extract Ⅱ was isolated using acetonitrile-water (30:70, *v*/*v*) as eluent at the flow rate of 2 mL/min to give compound **2a** (2.8 mg, *t*_R_ = 16.2 min) and compound **2a** (2.3 mg, *t*_R_ = 14.7 min). Fr. D of extract Ⅲ was isolated using acetonitrile-water (30:70, *v*/*v*) as eluent at the flow rate of 2 mL/min to give compound **3a** (2.0 mg, *t*_R_ = 17.5 min). Fr. E of extract Ⅳ was isolated using acetonitrile-water (50:50, *v*/*v*) as eluent at the flow rate of 2 mL/min to give compound **4a** (4.0 mg, *t*_R_ = 8.5 min).

4′,5-Dihydroxyflavanone 7-*O*-β-d-(4-*O*-methyl)glucopyranoside (**1a**): ^1^H- and ^13^C-NMR data see Table 1; (−)-HRESIMS *m*/*z* 447.1289 [M − H]^−^ (calcd. for C_22_H_23_O_10_, 447.1291).

5,7-Dihydroxyflavanone 4′-*O*-β-d-(4-*O*-methyl)glucopyranoside (**1b**): ^1^H- and ^13^C-NMR data see Table 1; (−)-HRESIMS *m*/*z* 447.1294 [M − H]^−^ (calcd. for C_22_H_23_O_10_, 447.1291).

5,7-Dihydroxyflavanone 4′-*O*-β-d-glucopyranoside (**1c**): ^1^H- and ^13^C-NMR data see Table 1; (−)-HRESIMS *m*/*z* 433.1122 [M − H]^−^ (calcd. for C_21_H_21_O_10_, 433.1135).

5-Hydroxyflavanone 4′,7-di-*O*-β-d-(4-*O*-methyl)glucopyranoside (**1d**): ^1^H- and ^13^C-NMR data see Table 1; (−)-HRESIMS *m*/*z* 623.1950 [M − H]^−^ (calcd. for C_29_H_35_O_15_, 623.1976).

4′,5,7-Trihydroxyflavone 3′-*O*-β-d-(4-*O*-methyl)glucopyranoside (**2a**): ^1^H- and ^13^C-NMR data see Table 2; (−)-HRESIMS *m*/*z* 461.1100 [M − H]^−^ (calcd. for C_22_H_21_O_11_, 461.1084).

3′,5,7-Trihydroxyflavone 4′-*O*-β-d-(4-*O*-methyl)glucopyranoside (**2b**): ^1^H- and ^13^C-NMR data see Table 2; (−)-HRESIMS *m*/*z* 461.1097 [M − H]^−^ (calcd. for C_22_H_21_O_11_, 461.1084).

5,7-Dihydroxy-4′-methoxyflavone-3′-*O*-β-d-(4-*O*-methyl)glucopyranoside (**3a**): ^1^H- and ^13^C-NMR data see Table 2; (−)-HRESIMS *m*/*z* 475.1261 [M − H]^−^ (calcd. for C_23_H_23_O_11_, 475.1240).

4′-methoxyisoflavone 7-*O*-β-d-(4-*O*-methyl)glucopyranoside (**4a**): ^1^H- and ^13^C-NMR data see Table 2; (−)-HRESIMS *m*/*z* 489.1422 [M − H]^−^ (calcd. for C_24_H_25_O_11_, 489.1397).

## Figures and Tables

**Table 1:** ^1^H- (600 MHz) and ^13^C-NMR (150 MHz) Data of **1a**, **1b**, **1c** and **1d** in DMSO-*d*_6._

No.	1a		1b		1c		1d	
	δ_C_, Type	δ_H_, Mult. (*J* in Hz)	δ_C_, Type	δ_H_, Mult. (*J* in Hz)	δ_C_, Type	δ_H_, Mult. (*J* in Hz)	δ_C_, Type	δ_H_, Mult. (*J* in Hz)
2	78.7, CH	5.50, brd (12.7)	78.0, CH	5.52, brd (12.2)	78.1, CH	5.52, brd (12.2)		
3	42.0, CH_2_	3.35, m 2.73, dt (17.4, 3.6)	42.0, CH_2_	3.27, m 2.73, dt (17.1, 3.2)		3.27, m 2.73, dt (17.1, 3.2)	42.1, CH_2_	3.36, m 2.80, dd (17.1, 2.8)
4	197.2, C		196.1, C		195.9, C		197.0, C	
5	162.9, C		163.5, C		163.5, C		162.9, C	
6	95.4, CH	6.15, brs	95.9, CH	5.89, brs	96.0, CH	5.87, brs	95.4, CH	6.17, brs
7	165.1, C		166.8, C		167.3, C		165.1, C	
8	96.5, CH	6.13, brs	95.0, CH	5.88, brs	95.2, CH	5.86, brs	96.5, CH	6.14, brs
9	162.8, C		162.8, C		162.7, C		162.6, C	
10	103.2, C		101.7, C		101.6, C		103.3, C	
1′	128.6, C		131.9, C		132.0, C		131.7, C	
2′	128.4, CH	7.33, d (8.2)	128.0, CH	7.43, d (8.2)	128.0, CH	7.43, d (8.2)	128.1, CH	7.45, d (8.2)
3′	115.2, CH	6.80, d (8.2)	116.1, CH	7.06, d (8.2)	116.2, CH	7.06, d (8.2)	116.2, CH	7.06, d (8.2)
4′	157.8, C		157.4, C		157.5, C		157.5, C	
5′	115.2, CH	6.80, d (8.2)	116.1, CH	7.06, d (8.2)	116.2, CH	7.06, d (8.2)	116.2, CH	7.06, d (8.2)
6′	128.4, CH	7.33, d (8.2)	128.0, CH	7.43, d (8.2)	128.0, CH	7.43, d (8.2)	128.1, CH	7.45, d (8.2)
1′′	99.1, CH	5.01, d (7.8)	99.9, CH	4.91, d (7.7)	100.3, CH	4.88, d (7.4)	99.1, CH	5.01, d (7.8)
2′′	73.2, CH	3.24, t (8.1)	73.4, CH	3.24, m	73.2, CH	3.24, m	73.2, CH	3.24, t (8.1)
3′′	76.0, CH	3.40, m	76.3, CH	3.41, m	77.1, CH	3.33, m	76.0, CH	3.40, m
4′′	78.8, CH	3.01, t (9.3)	79.0, CH	3.03, t (9.4)	69.7, CH	3.16, t (9.0)	78.8, CH	3.01, t (9.3)
5′′	75.6, CH	3.43, m	75.6, CH	3.38, m	76.6, CH	3.26, m	75.6, CH	3.43, m
6′′	60.1, CH_2_	3.60, brd (11.8) 3.47, m	60.2, CH_2_	3.63, dd (11.8, 4.4) 3.49, m	60.7, CH_2_	3.69, btd (11.8) 3.45, m	60.1, CH_2_	3.60, brd (11.8) 3.47, m
4′′-OCH_3_	59.6, CH_3_	3.44, s	59.7, CH_3_	3.45, s			59.6, CH_3_	3.44, s
1′′′							99.9, CH	4.91, d (7.7)
2′′′							73.4, CH	3.24, m
3′′′							76.3, CH	3.41, m
4′′′							79.0, CH	3.03, t (9.4)
5′′′							75.6, CH	3.38, m
6′′′							60.2, CH_2_	3.63, dd (11.8, 4.4) 3.49, m
4′′′-OCH_3_							59.7, CH_3_	3.45, s
5-OH		12.01, s		12.13, s		12.15, s		12.02, s
4′-OH		9.62, s						

**Table 2:** ^1^H- (600 MHz) and ^13^C-NMR (150 MHz) Data of **2a**, **2b**, **3a** and **4a** in DMSO-*d*_6._

No.	2a		2b		3a		4a	
	δ_C_, Type	δ_H_, Mult. (*J* in Hz)	δ_C_, Type	δ_H_, Mult. (*J* in Hz)	δ_C_, Type	δ_H_, Mult. (*J* in Hz)	δ_C_, Type	δ_H_, Mult. (*J* in Hz)
2	163.4, C		163.1, C		163.0, C		153.6, CH	8.44, s
3	103.0, CH	6.79, s	104.0, CH	6.82, s	103.7, CH	6.89, s	123.4, C	
4	181.7, C		181.7, C		181.7, C		174.6, C	
5	161.4, C		161.4, C		161.4, C		127.0, CH	8.06, d (8.8)
6	98.9, CH	6.18, brs	98.9, CH	6.20, brs	99.1, CH	6.17, brs	115.6, CH	7.15, dd (8.8, 1.8)
7	164.3, C		164.3, C		no show		161.3, C	
8	94.1, CH	6.49, brs	94.0, CH	6.50, brs	94.2, CH	6.49, brs	103.3, CH	7.24, d (1.8)
9	157.3, C		157.3, C		157.4, C		157.0, C	
10	103.6, C		103.8, C		103.4, C		118.5, C	
1′	121.1, C		124.7, C		122.9, C		124.0, C	
2′	114.4, C	7.74, d (2.2)	113.6, C	7.50, brs	112.8, C	7.70, d (2.0)	130.1, CH	7.53, d (8.4)
3′	145.7, C		146.9, C		146.5, C		115.6, CH	7.00, d (8.4)
4′	151.4, C		148.4, C		152.1, C		159.0, C	
5′	116.6, CH	6.95, d (8.5)	115.8, CH	7.22, d (8.5)	112.4, C	7.16, d (8.6)	115.6, CH	7.00, d (8.4)
6′	122.0, CH	7.64, dd (8.5, 2.2)	118.5, CH	7.51, brd (9.0)	121.0, CH	7.64, dd (8.6, 2.0)	130.1, CH	7.53, d (8.4)
1′′	101.5, CH	4.93, d (7.8)	100.7, CH	4.92, d (7.8)	99.4, CH	5.17, d (7.8)	99.6, CH	5.14, d (7.8)
2′′	73.5, CH	3.34, m	73.4, CH	3.34, m	73.4, CH	3.30, m	73.3, CH	3.30, m
3′′	75.8, CH	3.52, m	75.8, CH	3.52, m	76.6, CH	3.46, m	76.2, CH	3.46, m
4′′	79.3, CH	3.04, t (9.2)	79.0, CH	3.06, t (9.3)	79.1, CH	3.05, t (9.3)	78.9, CH	3.06, t (9.1)
5′′	75.7, CH	3.46, m	75.6, CH	3.46, m	75.6, CH	3.52, m	75.7, CH	3.52, m
6′′	60.4, CH_2_	3.70, brs (11.4) 3.56, m	60.2, CH_2_	3.66, brs (11.4) 3.52, m	60.2, CH_2_	3.64, brs (11.0) 3.52, m	60.2, CH_2_	3.66, brs (11.0) 3.52, m
4′-OCH_3_					55.8, CH_3_	3.86, s	55.2, CH_3_	3.79, s
4′′-OCH_3_	59.7, CH_3_	3.47, s	59.7, CH_3_	3.47, s	59.7, CH_3_	3.47, s	59.7, CH_3_	3.47, s
5-OH		12.96, s		12.90, s		12.90, s		

## Supplementary Materials

The supplementary materials are available online.
