# Supplementary material for: Microbial Transformation of Flavonoids by Isaria fumosorosea ACCC 37814
Source: Molecules. 2019 Mar 15;24(6):1028. doi: 10.3390/molecules24061028 (PMC6471136; doi:10.3390/molecules24061028)
Supplement: Supplementary file 1 [file molecules-24-01028-s001.pdf]

SUPPLEMENTARY INFORMATION FOR

Microbial transformation of flavonoids by *Isaria fumosorosea* ACCC 37814

Fangmin Dou, Zhi Wang, Guiying Li, Baoqing Dun*

The National Key Facility for Crop Gene Resources and Genetic Improvement, Institute of Crop Sciences, Chinese Academy of Agricultural Sciences, 12 Zhongguancun South Street, Beijing 100081, P.R. China

*Corresponding author: B. Dun, Tel: +86-10-8210-8746, Email: dunbaoqing@caas.cn

Contents

Table S1. The biotransformation of phenolic substrates by *I. fumosorosea* ACCC 37814..... 4

Figure S1 HRESIMS spectra 6

Figure S1.1 (−)-HRESIMS spectrum of compound **1a** 6

Figure S1.2 (−)-HRESIMS spectrum of compound **1b** 6

Figure S1.3 (−)-HRESIMS spectrum of compound **1c** 7

Figure S1.4 (−)-HRESIMS spectrum of compound **1d** 7

Figure S1.5 (−)-HRESIMS spectrum of compound **2a** 8

Figure S1.6 (−)-HRESIMS spectrum of compound **2b** 8

Figure S1.7 (−)-HRESIMS spectrum of compound **3a** 9

Figure S1.8 (−)-HRESIMS spectrum of compound **4a** 9

Figure S2 NMR spectra 10

Figure S2.1 ^{1}H NMR spectrum of compound **1a** in DMSO-d_6 10

Figure S2.2 ^{13}C NMR spectrum of compound **1a** in DMSO-d_6 11

Figure S2.3 HSQC spectrum of compound **1a** in DMSO-d_6 12

Figure S2.4 HMBC spectrum of compound **1a** in DMSO-d_6 13

Figure S2.5 ^{1}H NMR spectrum of compound **1b** in DMSO-d_6 14

Figure S2.6 ^{13}C NMR spectrum of compound **1b** in DMSO-d_6 15

Figure S2.7 HSQC spectrum of compound **1b** in DMSO-d_6 16

Figure S2.8 HMBC spectrum of compound **1b** in DMSO-d_6 17

Figure S2.9 ^{1}H NMR spectrum of compound **1c** in DMSO-d_6 18

Figure S2.10 ^{13}C NMR spectrum of compound **1c** in DMSO-d_6 19

Figure S2.11 HSQC spectrum of compound **1c** in DMSO-d_6 20

Figure S2.12 HMBC spectrum of compound **1c** in DMSO-d_6 21

Figure S2.13 ^{1}H NMR spectrum of compound **1d** in DMSO-d_6 22

Figure S2.14 ^{13}C NMR spectrum of compound **1d** in DMSO-d_6 23

Figure S2.15 HSQC spectrum of compound **1d** in DMSO-d_6 24

Figure S2.16 HMBC spectrum of compound **1d** in DMSO-d_6 25

Figure S2.17 ^{1}H NMR spectrum of compound **2a** in DMSO-d_6 26

Figure S2.18 ^{13}C NMR spectrum of compound **2a** in DMSO-d_6 27

Figure S2.19 HSQC spectrum of compound **2a** in DMSO-d_6 28

Figure S2.20 HMBC spectrum of compound **2a** in DMSO-d_6 29

Figure S2.21 ^{1}H NMR spectrum of compound **2b** in DMSO-d_6 30

Figure S2.22 ^{13}C NMR spectrum of compound **2b** in DMSO-d_6 31

Figure S2.23 HSQC spectrum of compound **2b** in DMSO-d_6 32

Figure S2.24 HMBC spectrum of compound **2b** in DMSO-d_6 33

Figure S2.25 ^{1}H NMR spectrum of compound **3a** in DMSO-d_6 34

Figure S2.26 ^{13}C NMR spectrum of compound **3a** in DMSO-d_6 35

Figure S2.27 HSQC spectrum of compound **3a** in DMSO-d_6 36

Figure S2.28 HMBC spectrum of compound **3a** in DMSO-d_6 37

Figure S2.29 ^{1}H NMR spectrum of compound **4a** in DMSO-d_6 38

Figure S2.30 ^{13}C NMR spectrum of compound **4a** in DMSO-d_6 39

Figure S2.31 HMBC spectrum of compound **4a** in DMSO-d_6 40

Table S1. The biotransformation of phenolic substrates by *I. fumosorosea* ACCC 37814.

Substrate	Chemical structure	Unreacted Substrate [%]	Mono-Glc [%]	Mono-MeGlcA [%]	Mono-MeGlcB [%]	Di-MeGlc [%]
Naringenin **1**	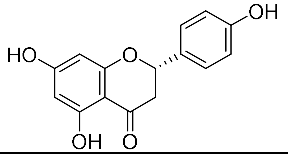	7.3 ± 1.0	13.6 ± 1.4	30.9 ± 3.8	39.9 ± 0.6	8.3 ± 1.9
Luteolin **2**	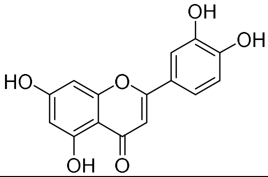	62.6 ± 4.6	NA	12.8 ± 3.7	24.6 ± 8.3	NA
Diosmetin **3**	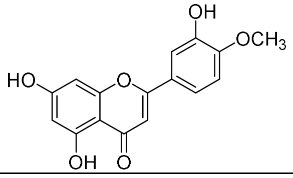	31.8 ± 0.2	NA	68.2 ± 0.2	NA	NA
Formononetin **4**	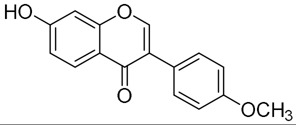	78.2 ± 0.1	NA	21.8 ± 0.1	NA	NA
Kaempferol **5**	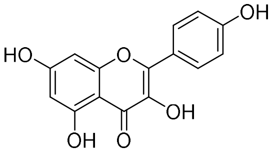	2.5 ± 0.9	24.3 ± 1.5	58.7 ± 2.8	14.5 ± 0.3	NA
Hesperetin **6**	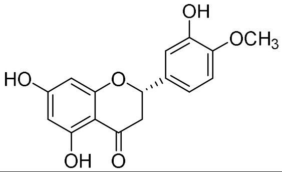	5.3 ± 0.4	12.5 ± 0.6	82.2 ± 1.5	NA	NA
Apigenin **7**	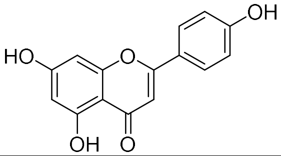	3.5 ± 3.1	NA	96.5 ± 2.9	NA	NA
Genistein **8**	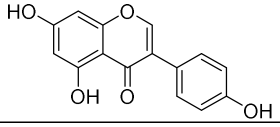	0.6 ± 0.6	0.3 ± 0.1	95.1 ± 0.4	4.0 ± 0.2	NA
Emodin **9**	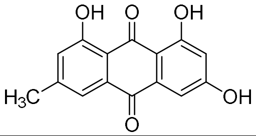	33.2 ± 4.0	NA	66.8 ± 14.7	5.8 ± 1.8	NA
Curcumin **10**	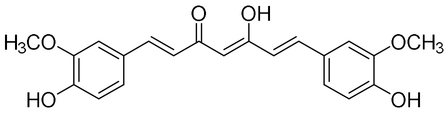	NA	NA	NA	NA	NA

The substrate-to-product conversion rate [%] of individual product was calculated from the HPLC peak area (recorded at 300 nm) of this product divided by the area sum of all products and the unreacted substrate. Values represent the mean ± SD from three independent experiments of three replicates each. Mono-Glc: mono-glucoside; Mono-MeGlcA and –B: mono-(4-*O*-methyl)glucoside regioisomers; Di-MeGlc: di-(4-*O*-methyl)glucoside. NA, not applicable. No detectable glycosylated product of curcumin **10** was found in the biotransformation culture of *I. fumosorosea* ACCC 37814.

Figure S1 HRESIMS spectra

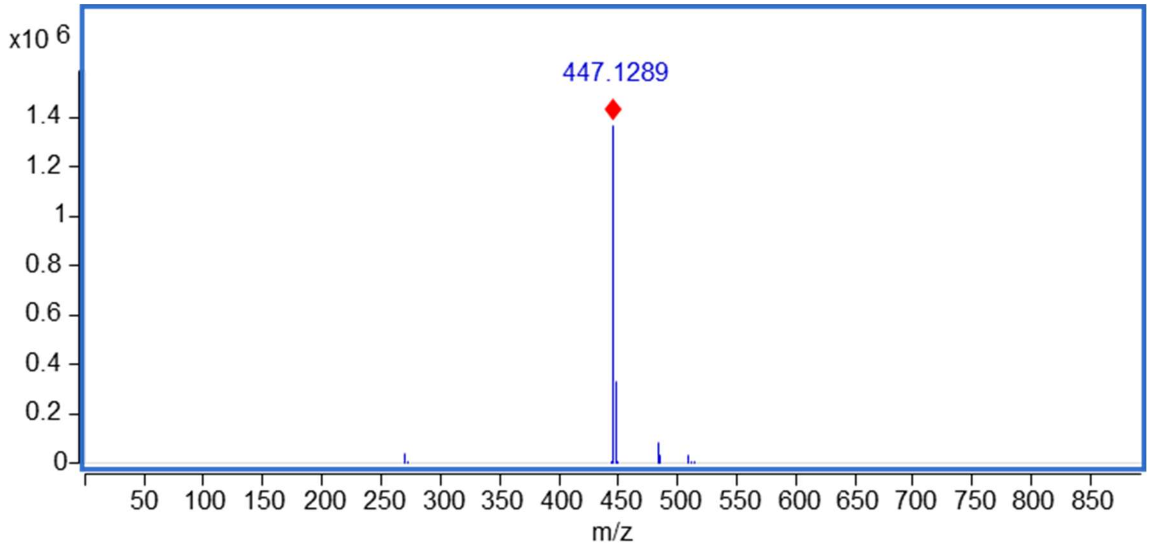

Figure S1.1 (−)-HRESIMS spectrum of compound **1a**

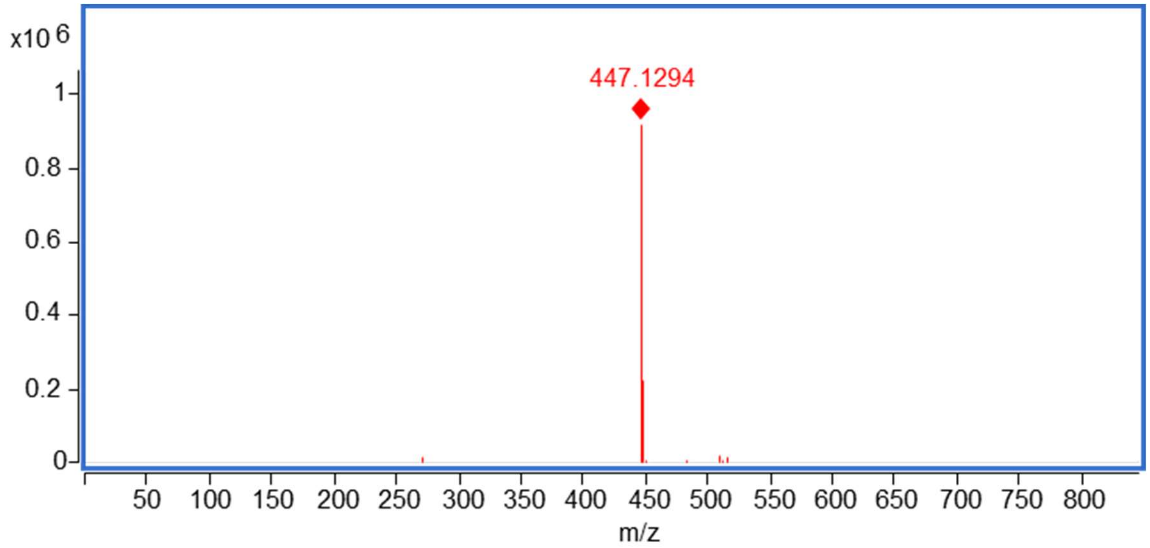

Figure S1.2 (−)-HRESIMS spectrum of compound **1b**

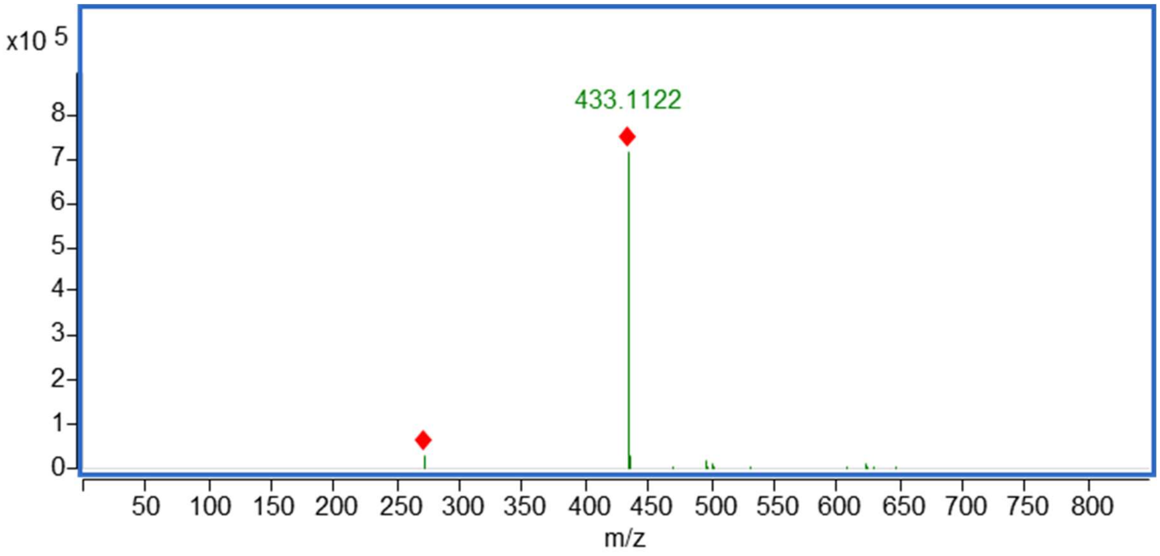

Figure S1.3 (−)-HRESIMS spectrum of compound **1c**

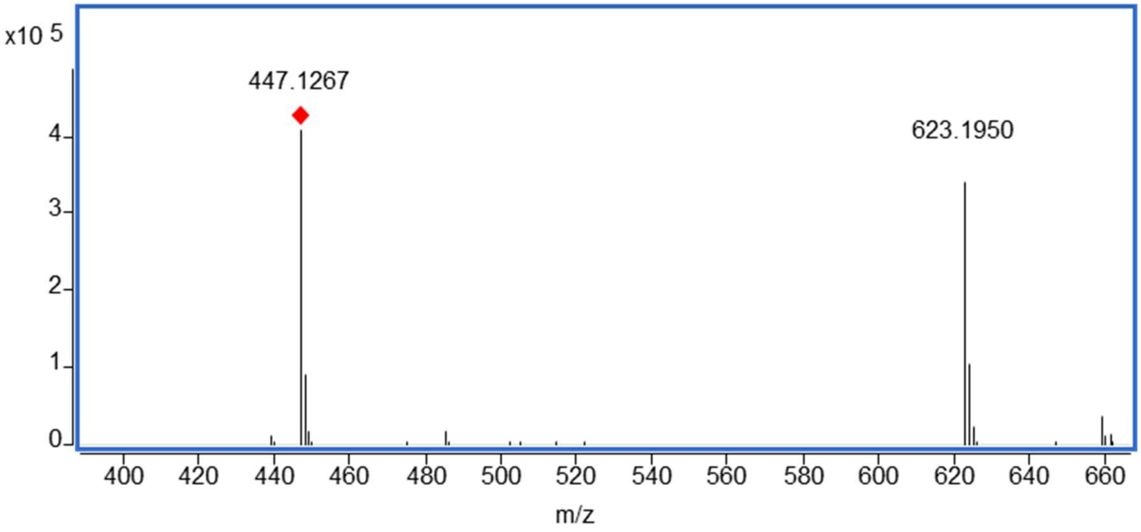

Figure S1.4 (−)-HRESIMS spectrum of compound **1d**

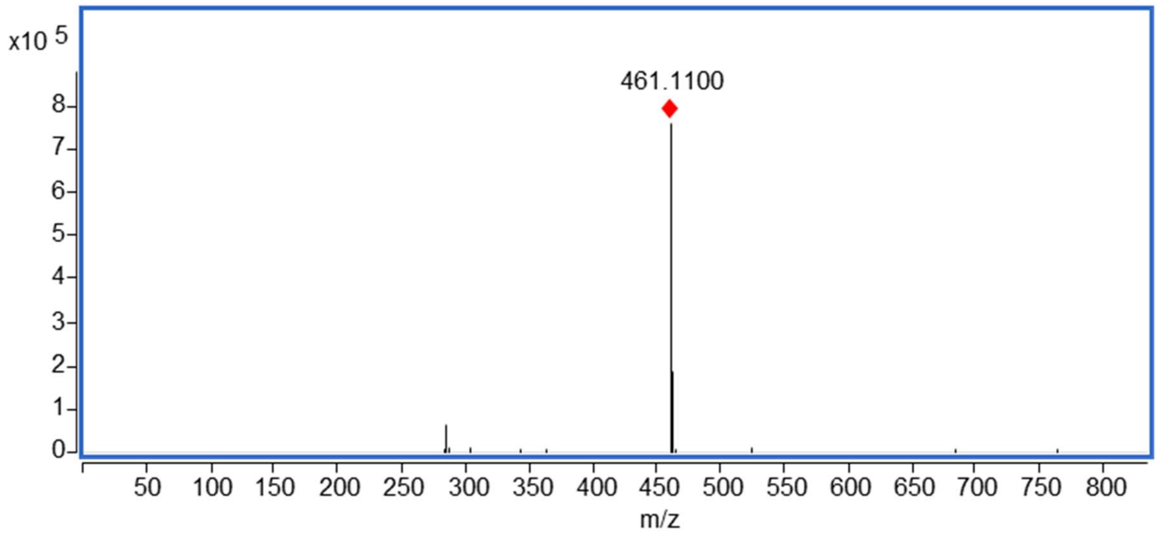

Figure S1.5 (−)-HRESIMS spectrum of compound **2a**

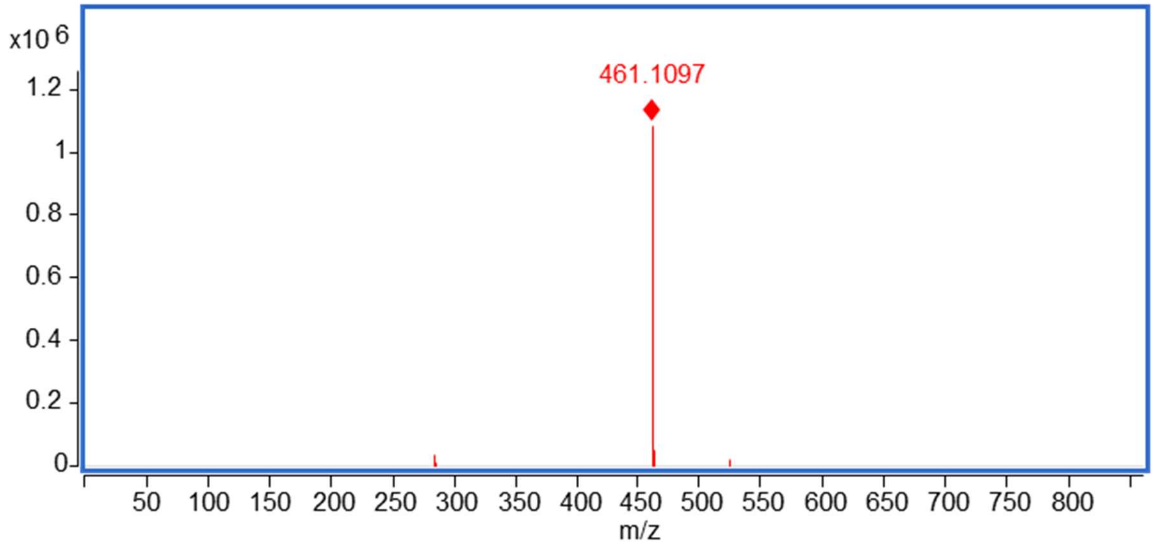

Figure S1.6 (−)-HRESIMS spectrum of compound **2b**

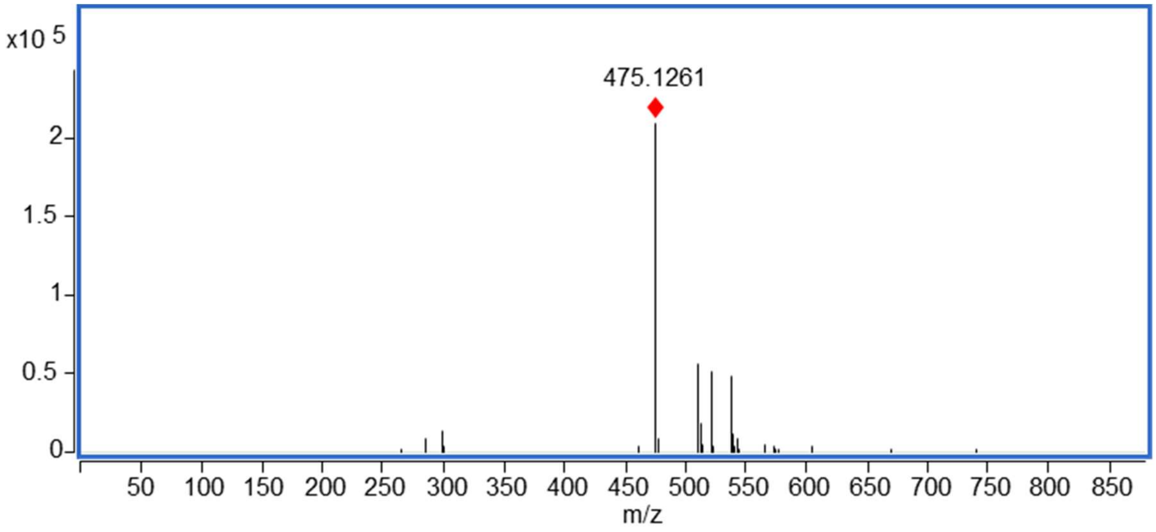

Figure S1.7 (−)-HRESIMS spectrum of compound **3a**

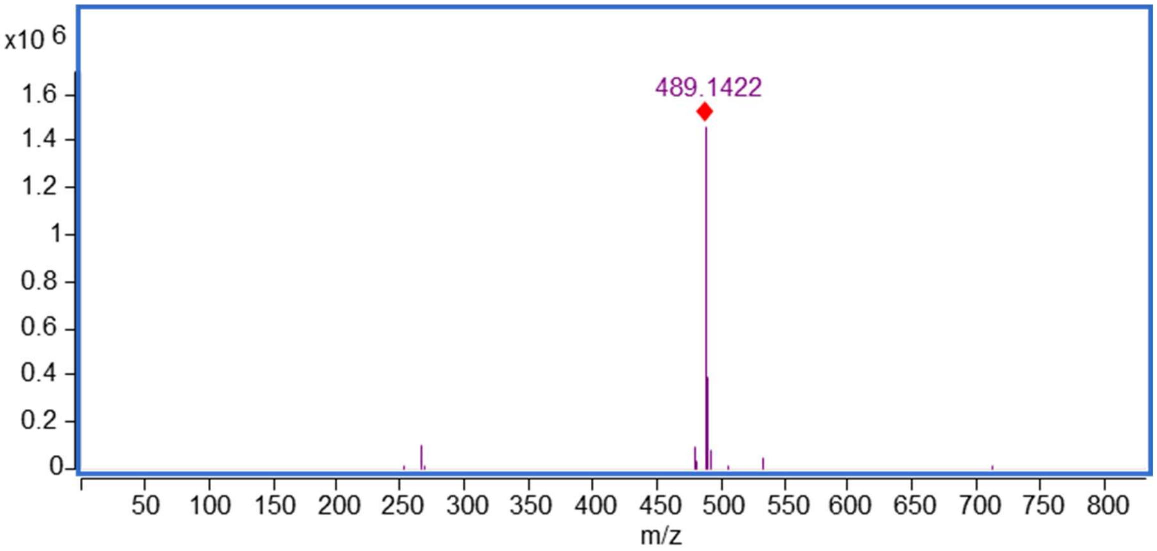

Figure S1.8 (−)-HRESIMS spectrum of compound **4a**

Figure S2 NMR spectra

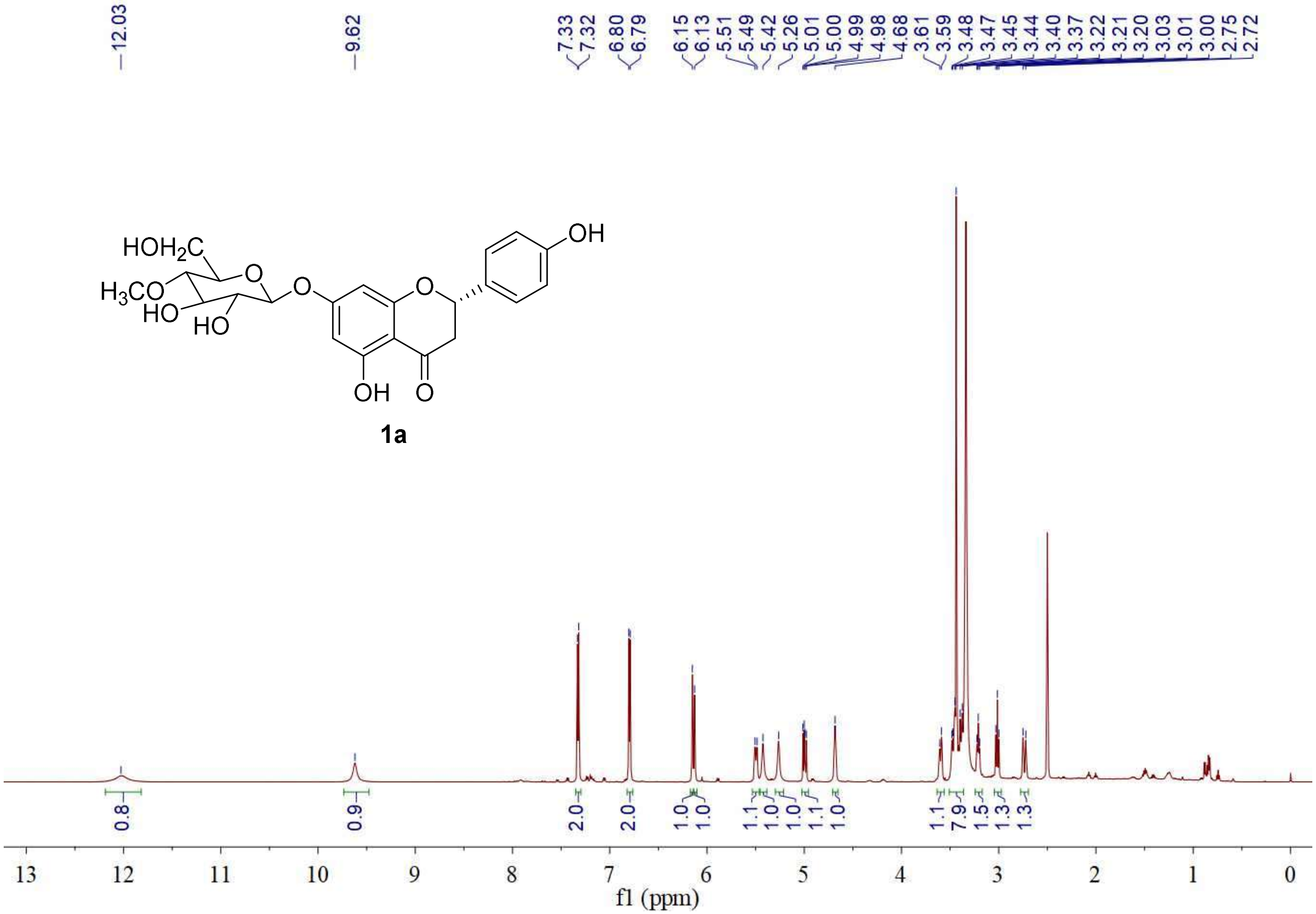

Figure S2.1 ^{1}H NMR spectrum of compound **1a** in DMSO-d_6

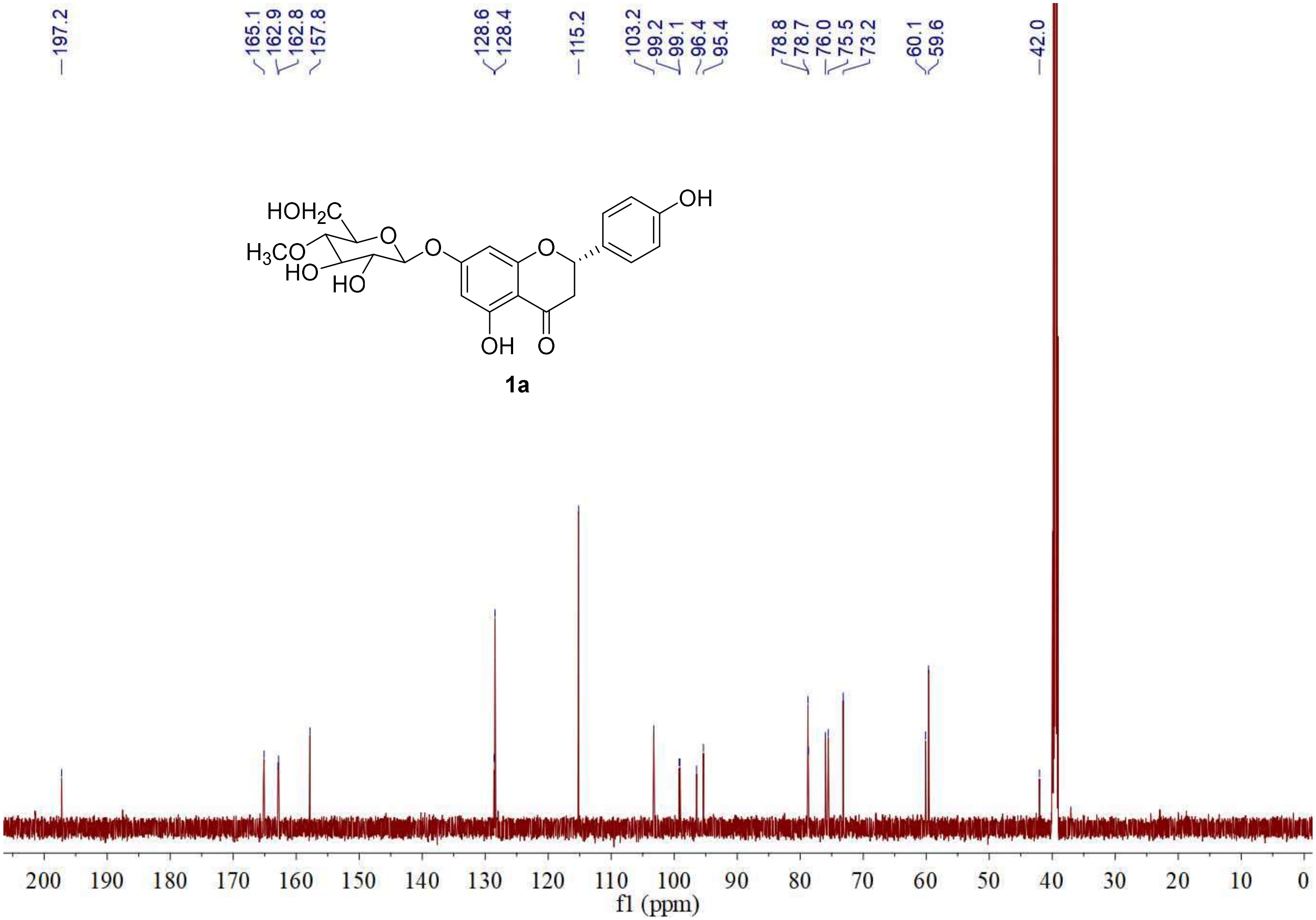

Figure S2.2 ^{13}C NMR spectrum of compound **1a** in DMSO-d_6

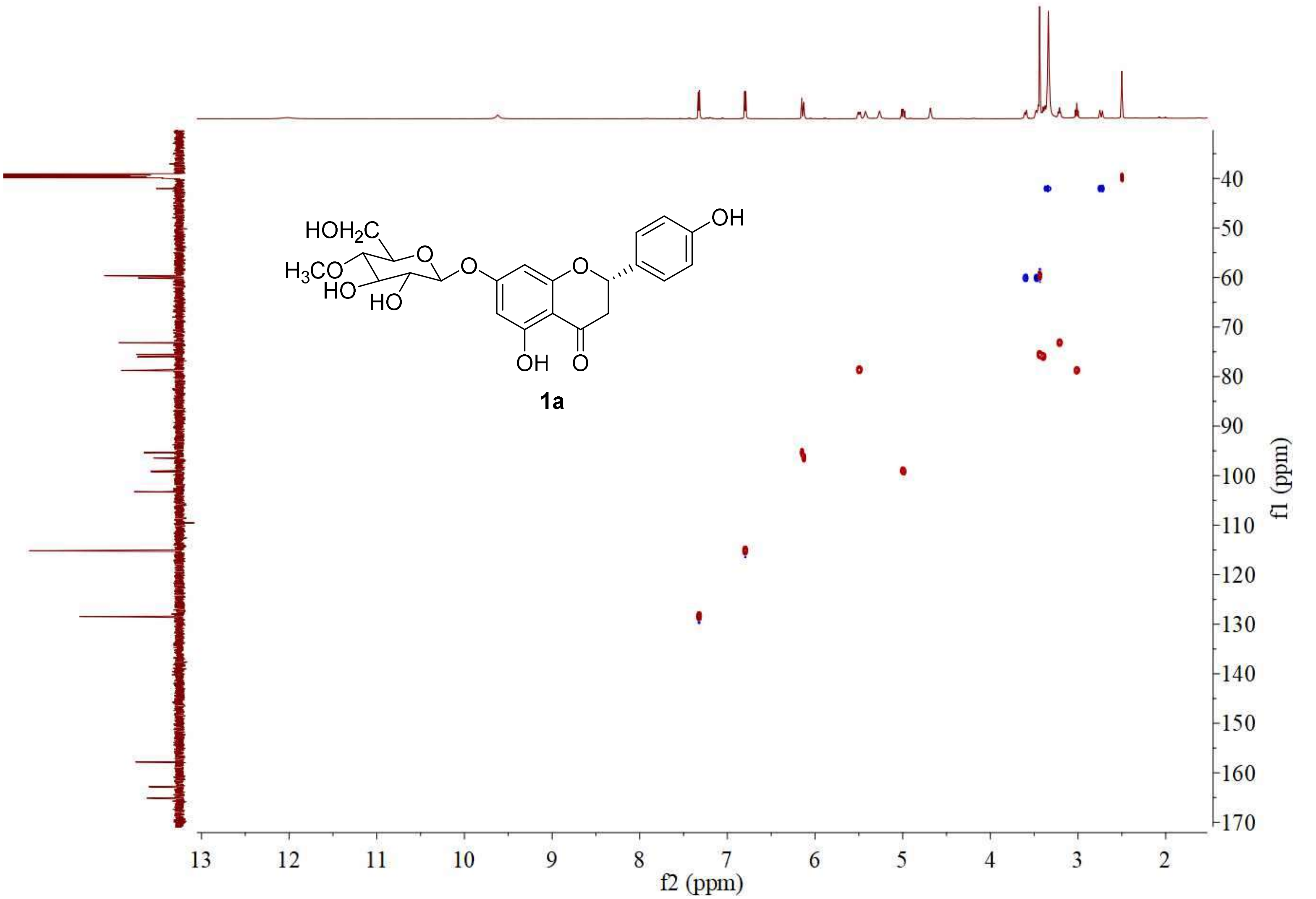

Figure S2.3 HSQC spectrum of compound **1a** in DMSO-d_6

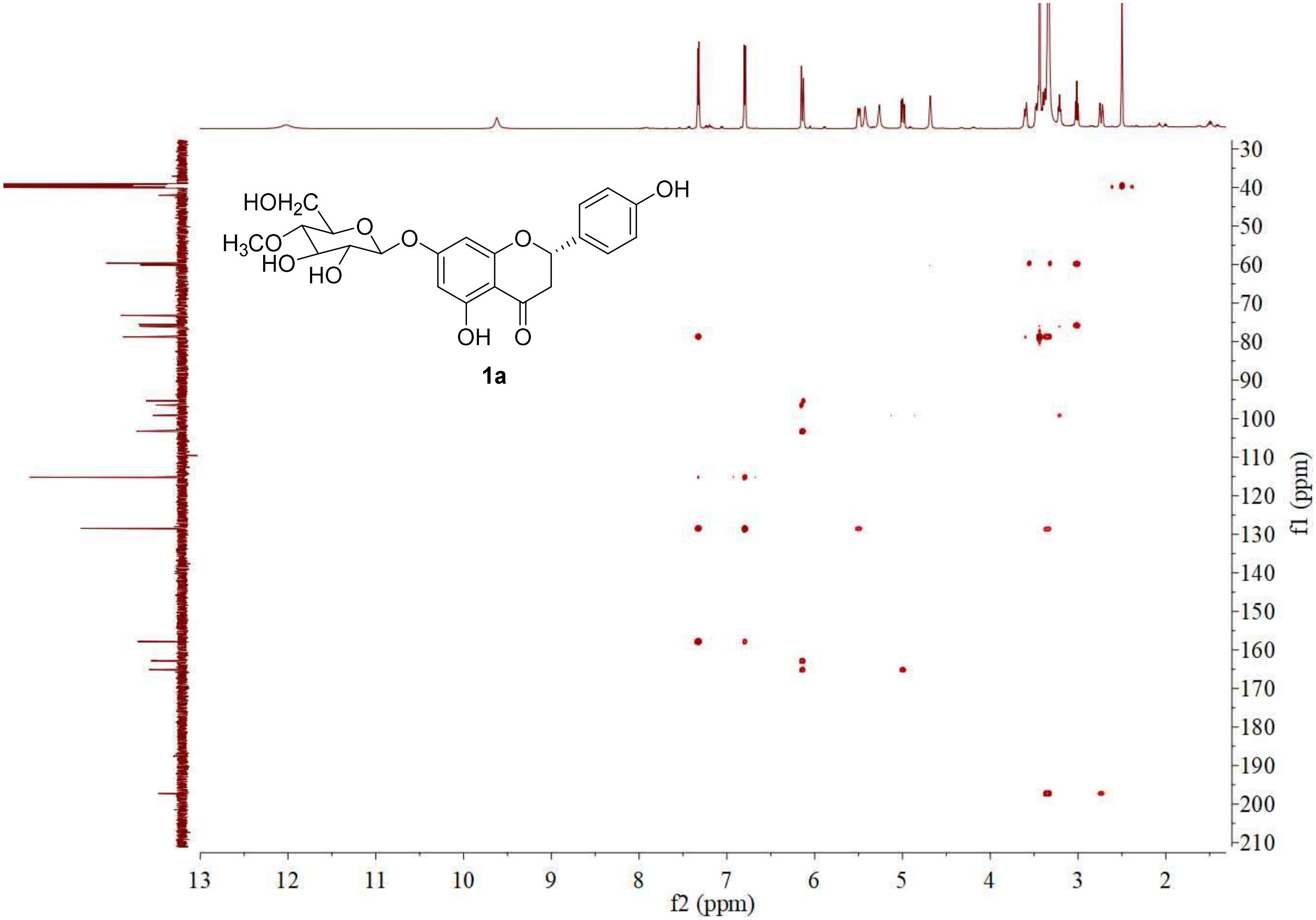

Figure S2.4 HMBC spectrum of compound **1a** in DMSO-d_6

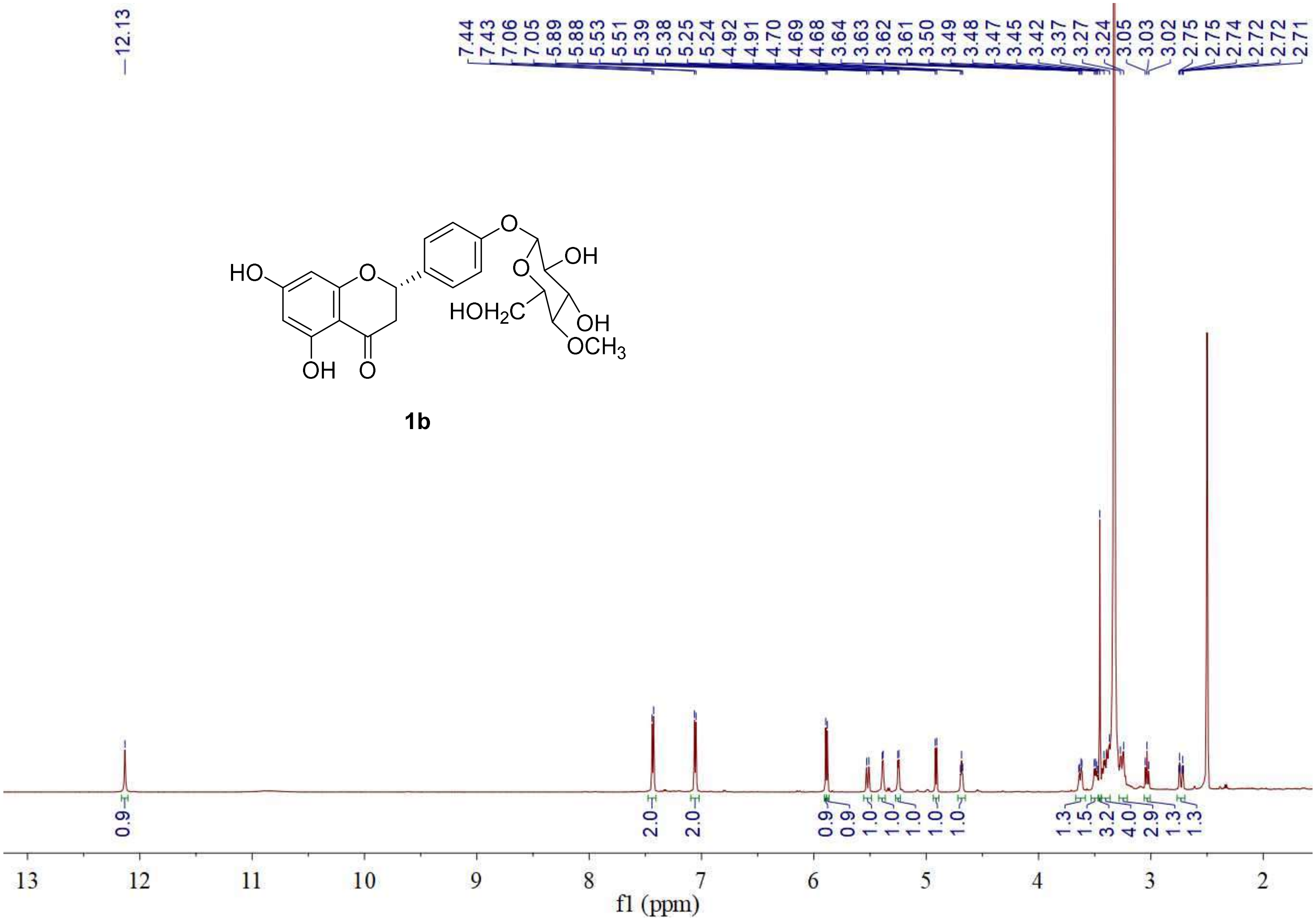

Figure S2.5 ^{1}H NMR spectrum of compound **1b** in DMSO-d_6

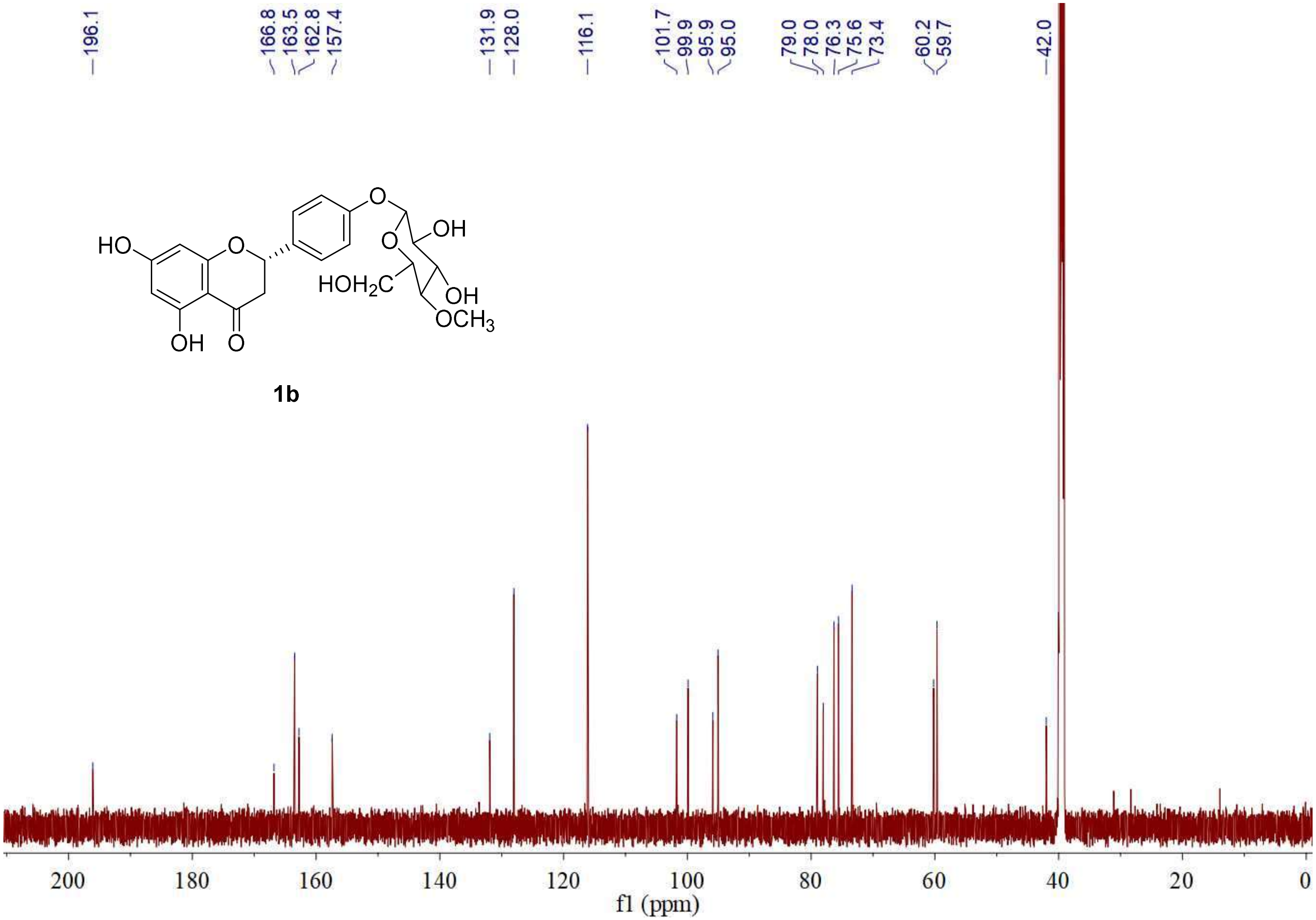

Figure S2.6 ^{13}C NMR spectrum of compound **1b** in DMSO-d_6

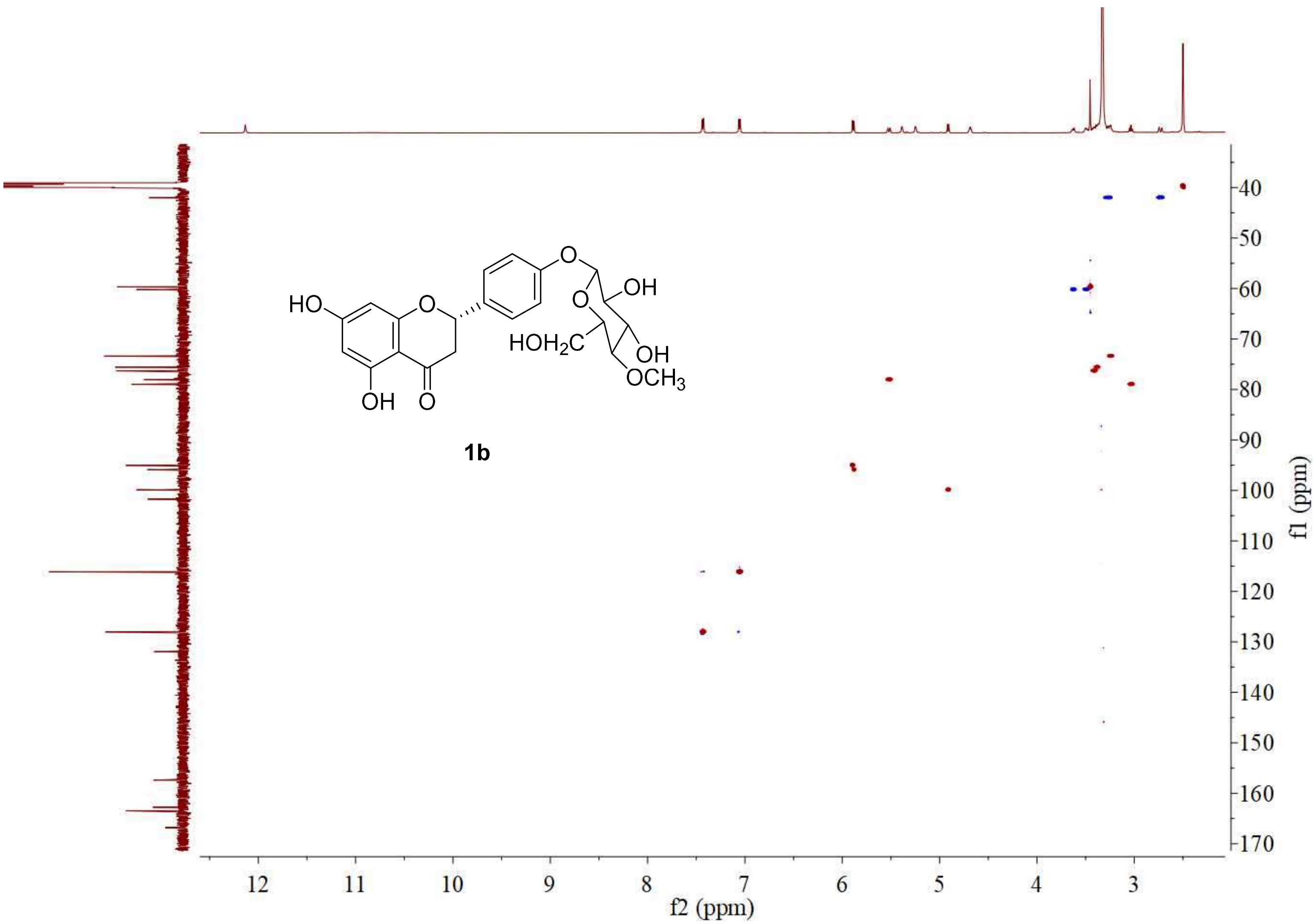

Figure S2.7 HSQC spectrum of compound **1b** in DMSO-d_6

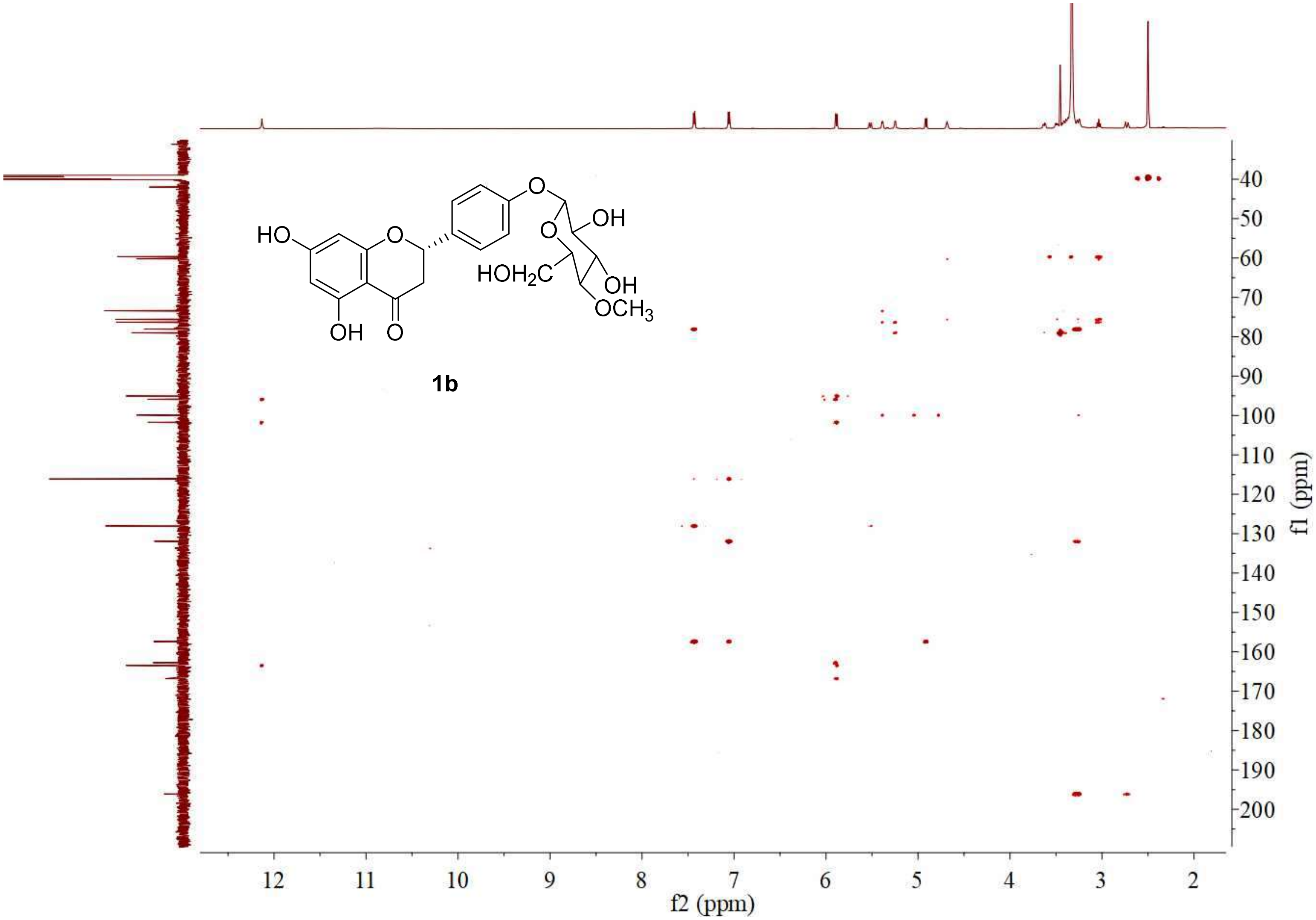

Figure S2.8 HMBC spectrum of compound **1b** in DMSO-d_6

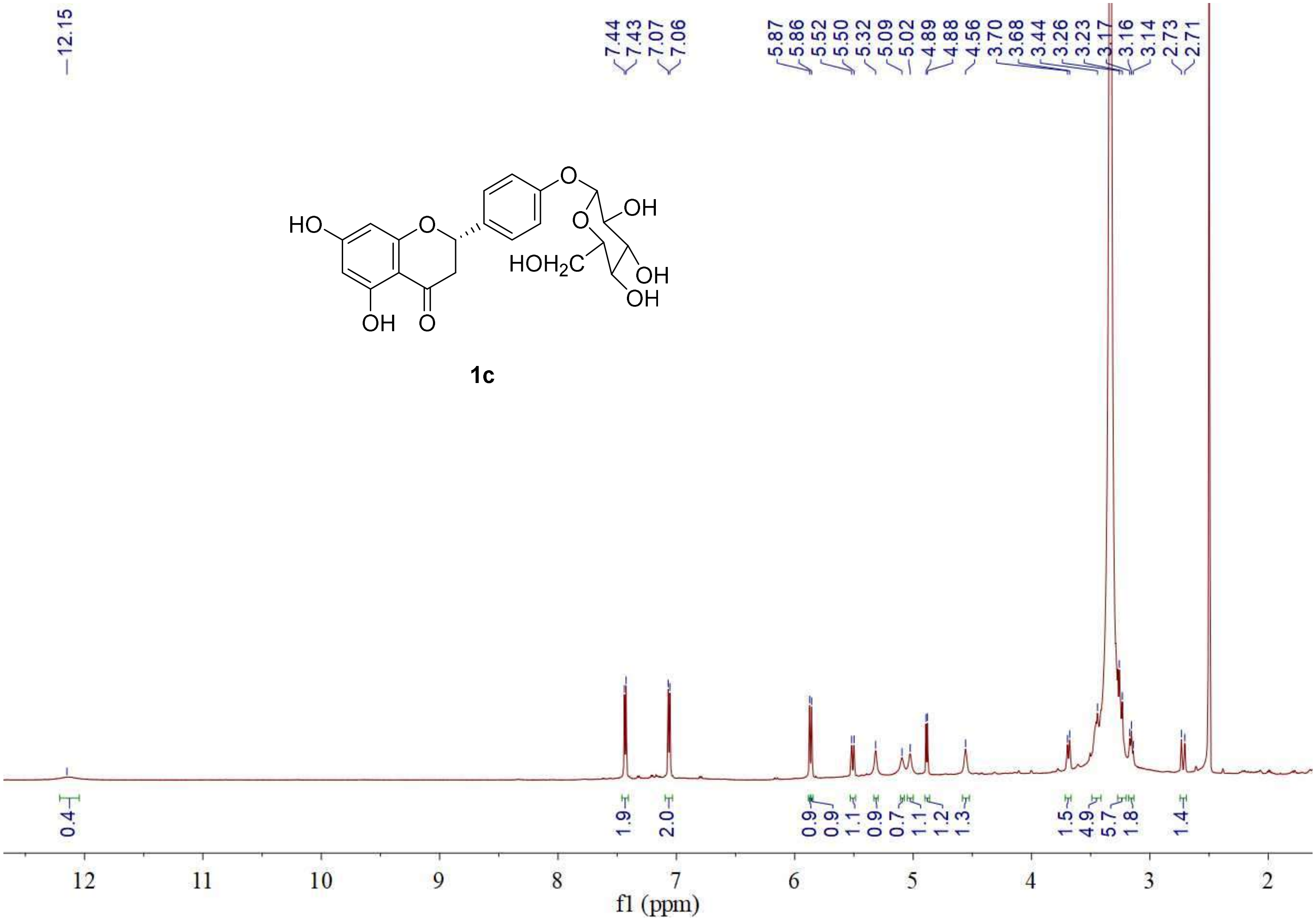

Figure S2.9 ^{1}H NMR spectrum of compound **1c** in DMSO-d_6

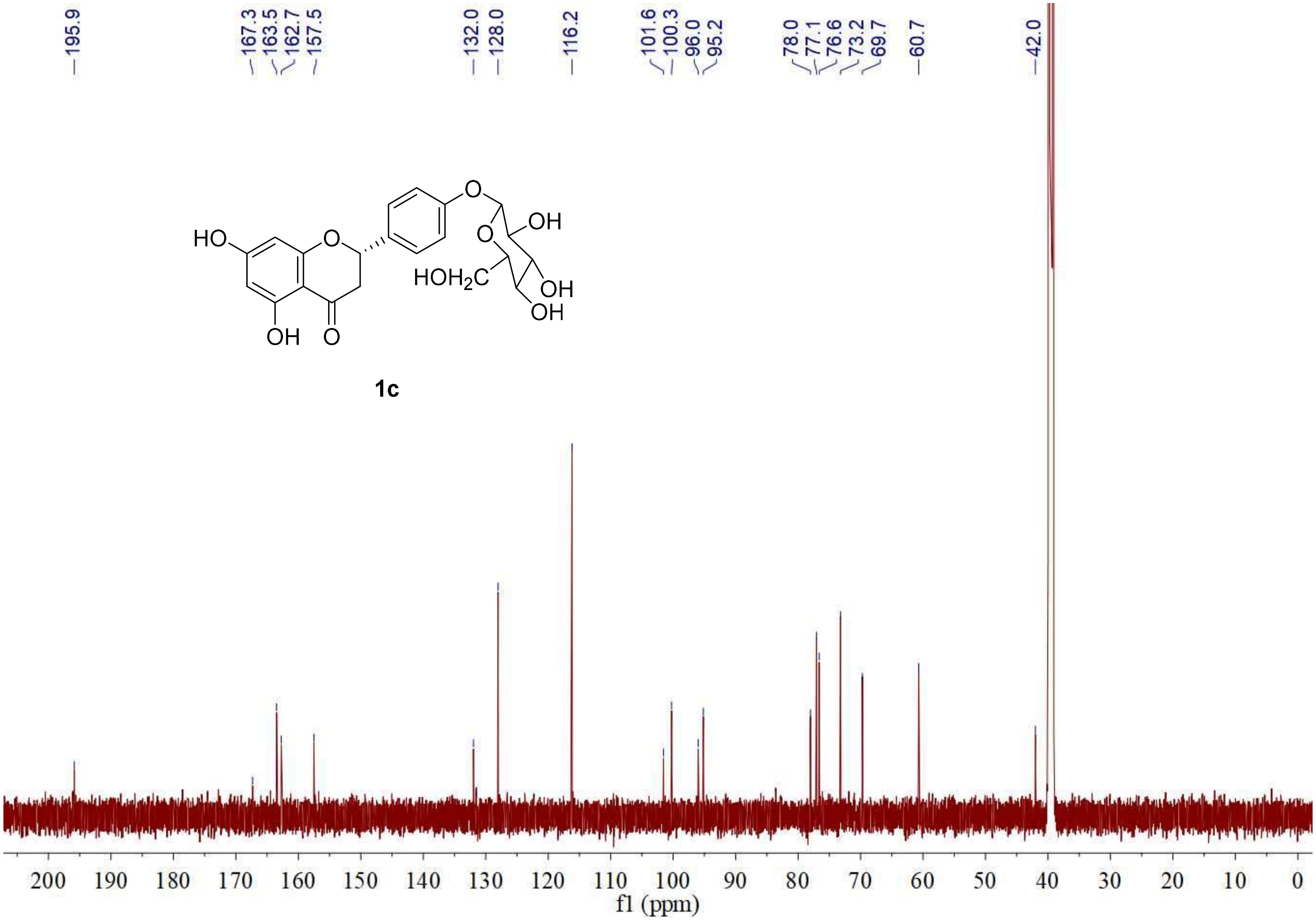

Figure S2.10 ^{13}C NMR spectrum of compound **1c** in DMSO-d_6

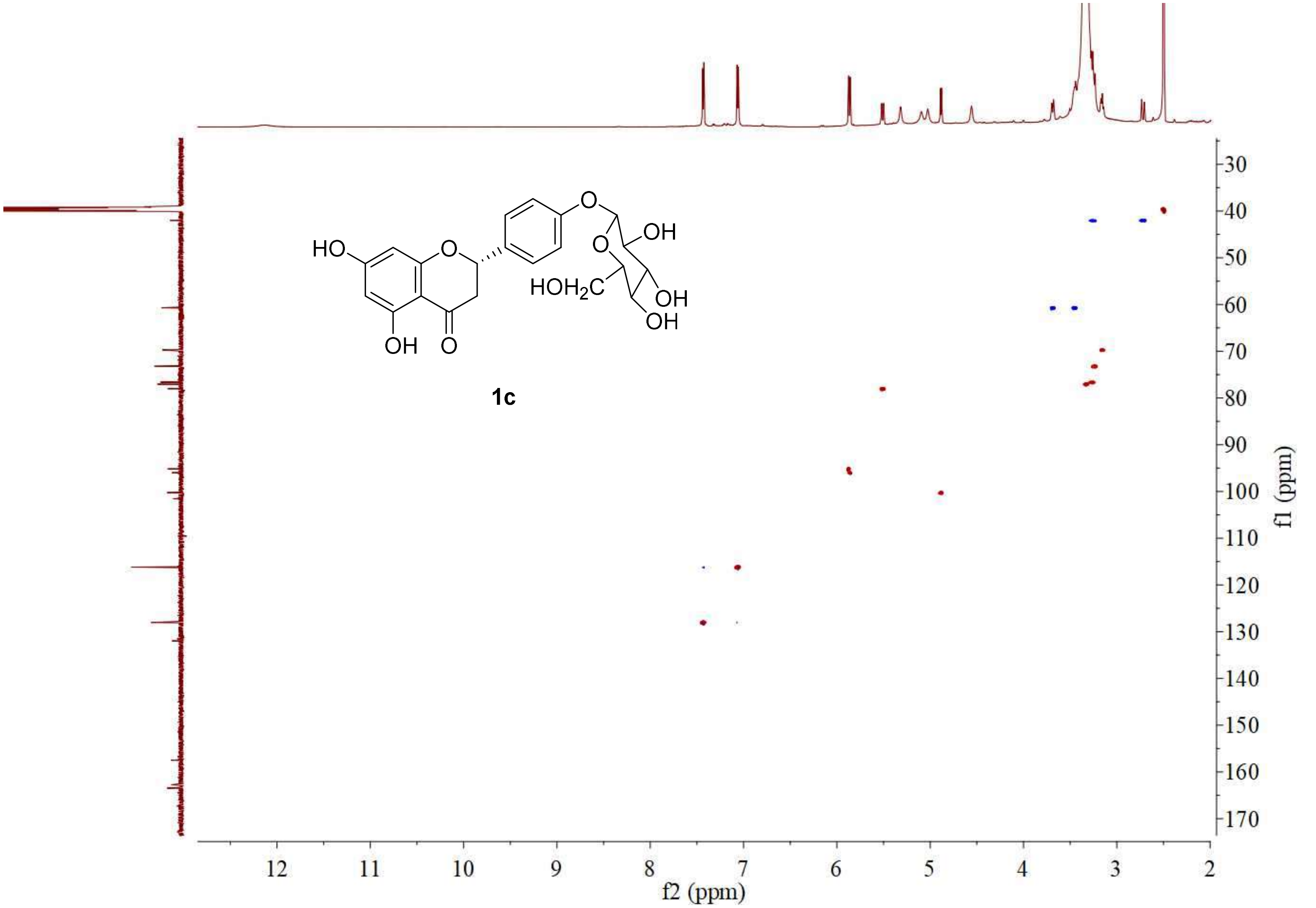

Figure S2.11 HSQC spectrum of compound **1c** in DMSO-d_6

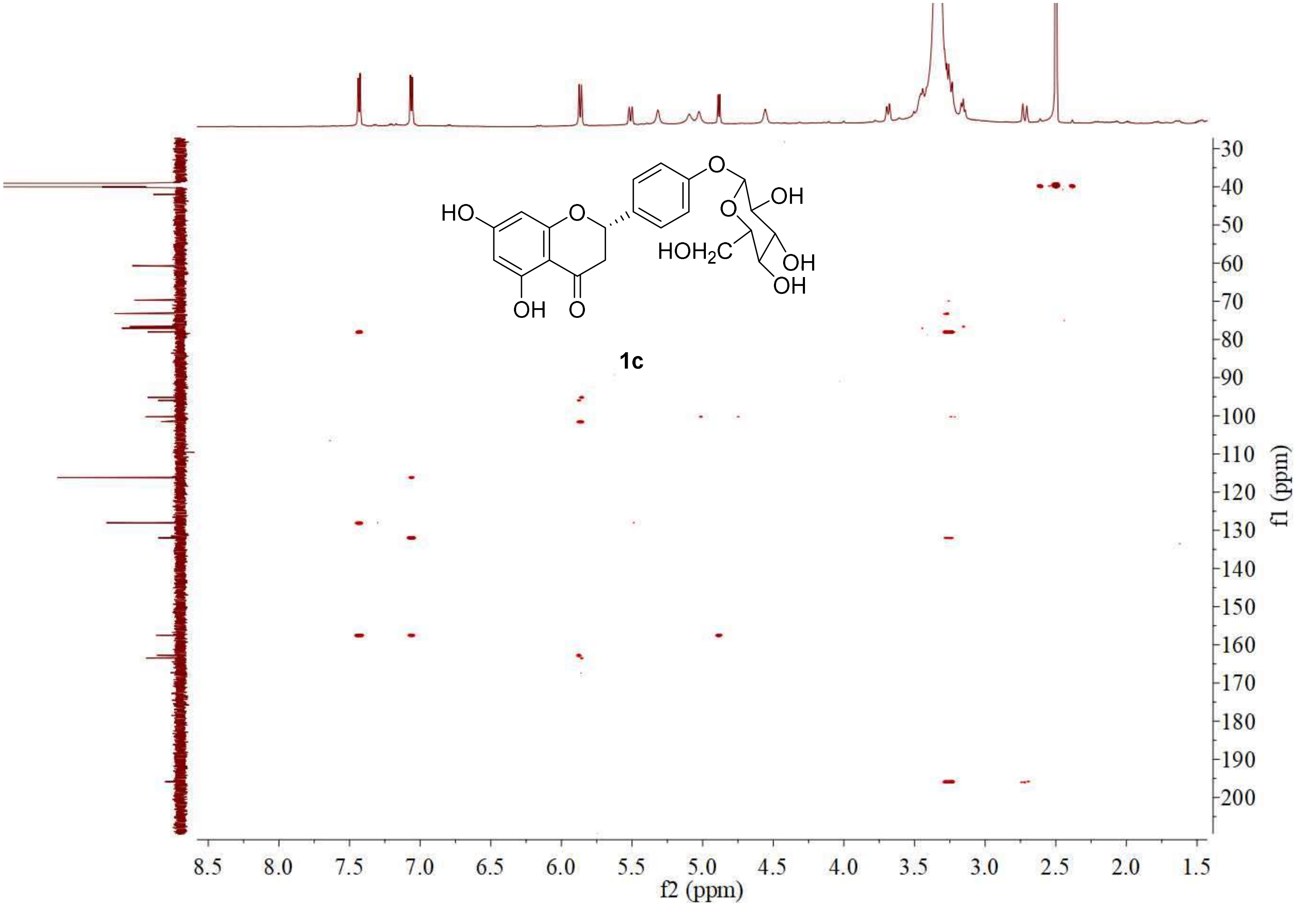

Figure S2.12 HMBC spectrum of compound **1c** in DMSO-d_6

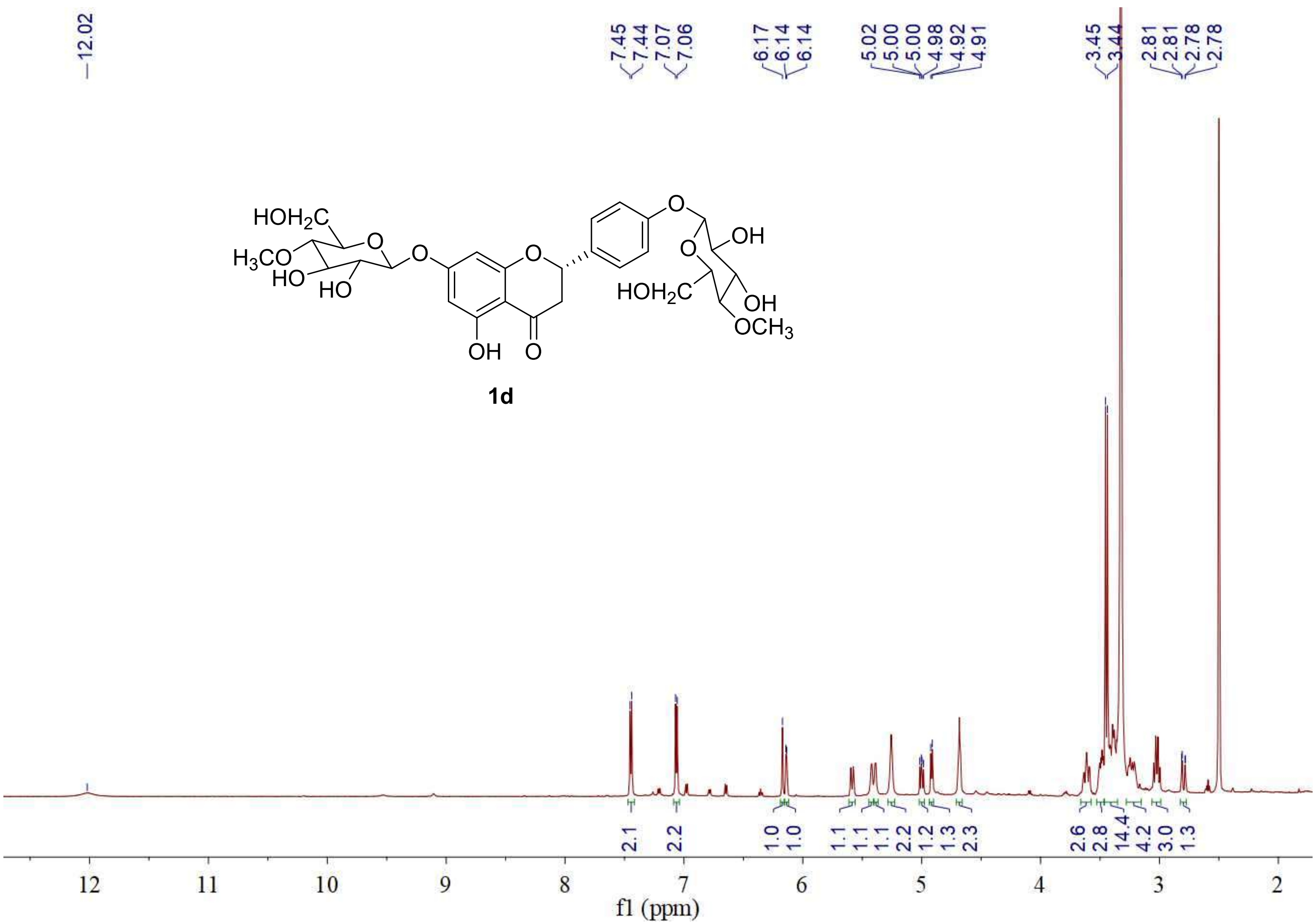

Figure S2.13 ^{1}H NMR spectrum of compound **1d** in DMSO-d_6

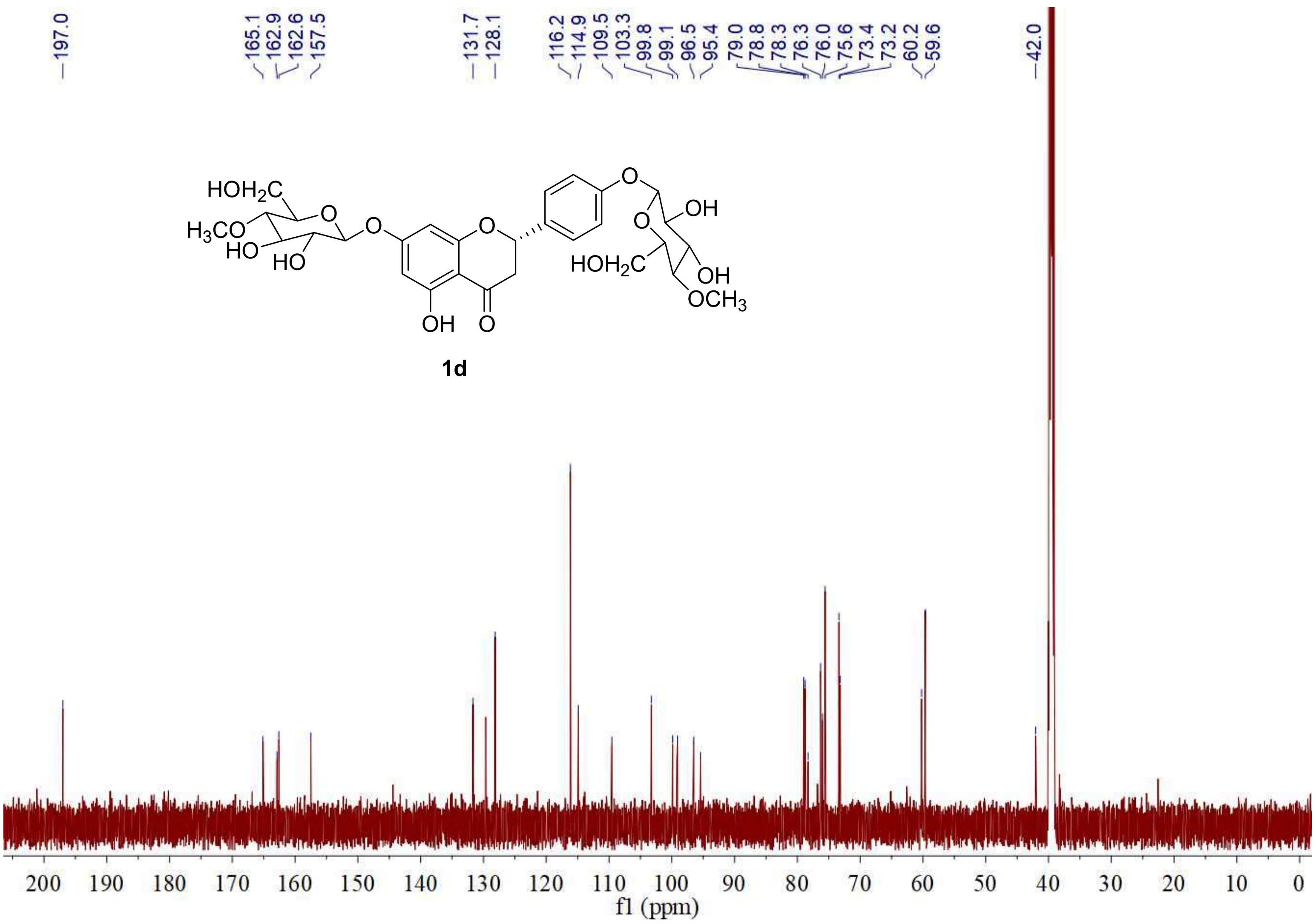

Figure S2.14 ^{13}C NMR spectrum of compound **1d** in DMSO-d_6

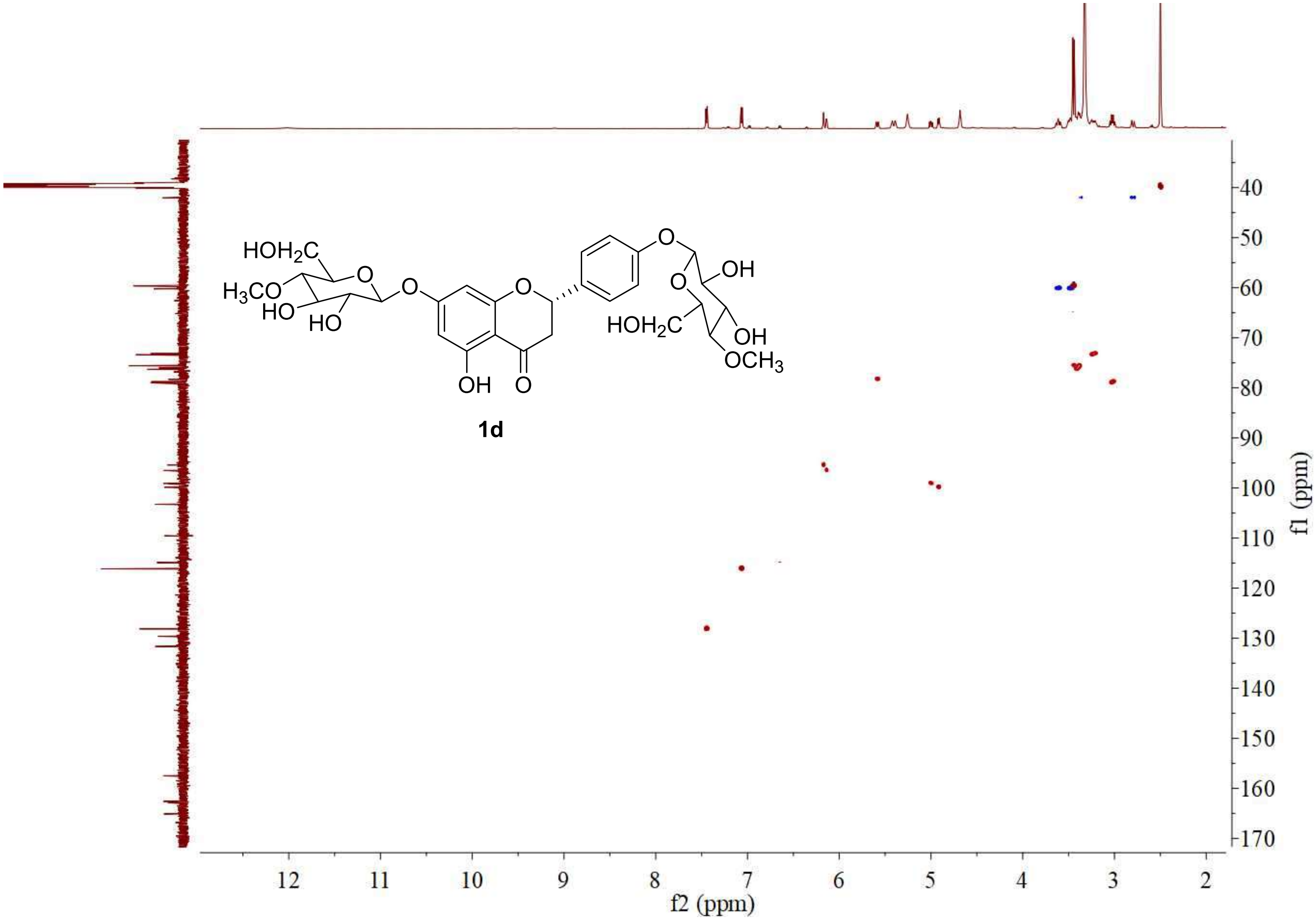

Figure S2.15 HSQC spectrum of compound **1d** in DMSO-d_6

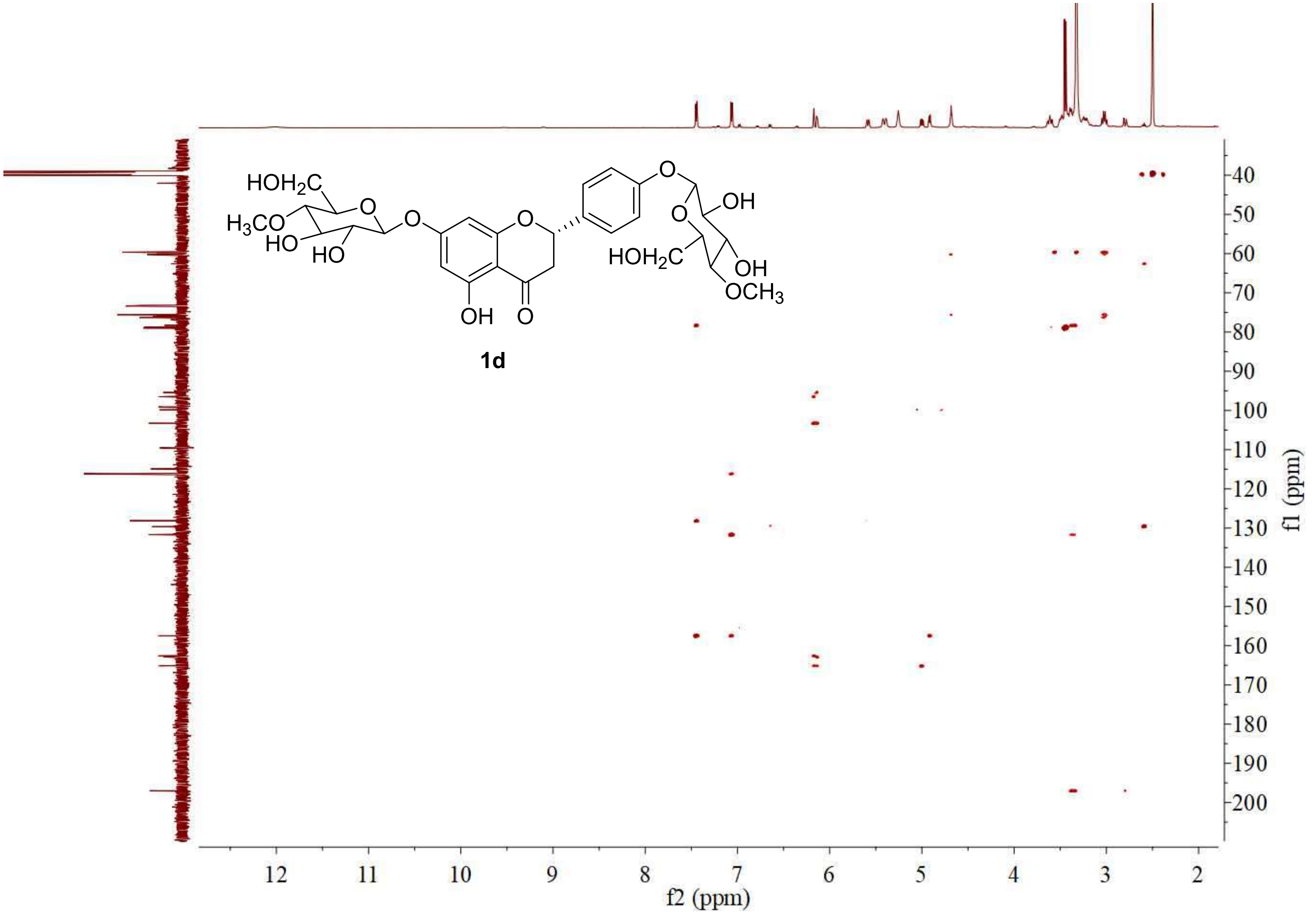

Figure S2.16 HMBC spectrum of compound **1d** in DMSO-d_6

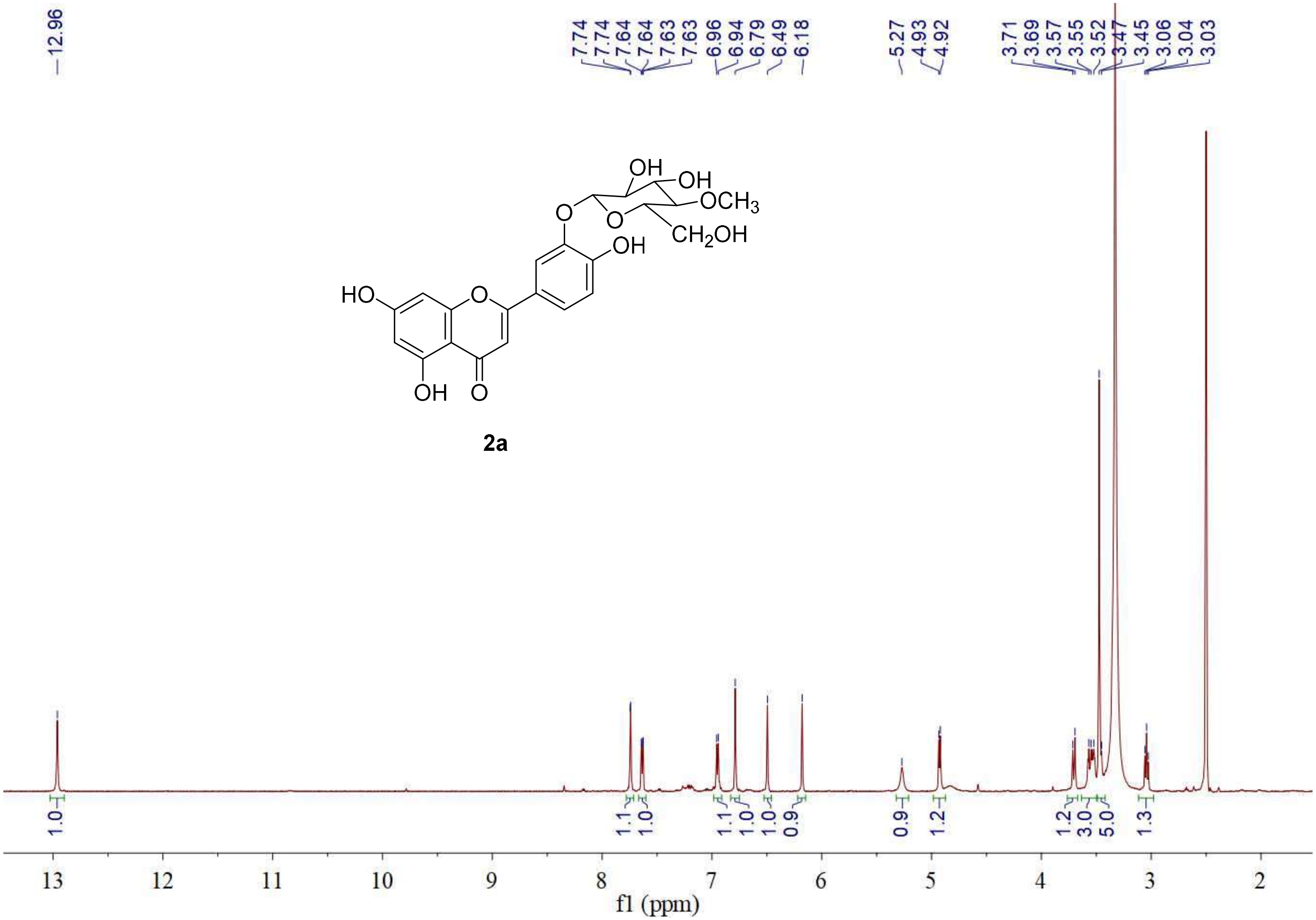

Figure S2.17 ^{1}H NMR spectrum of compound **2a** in DMSO-d_6

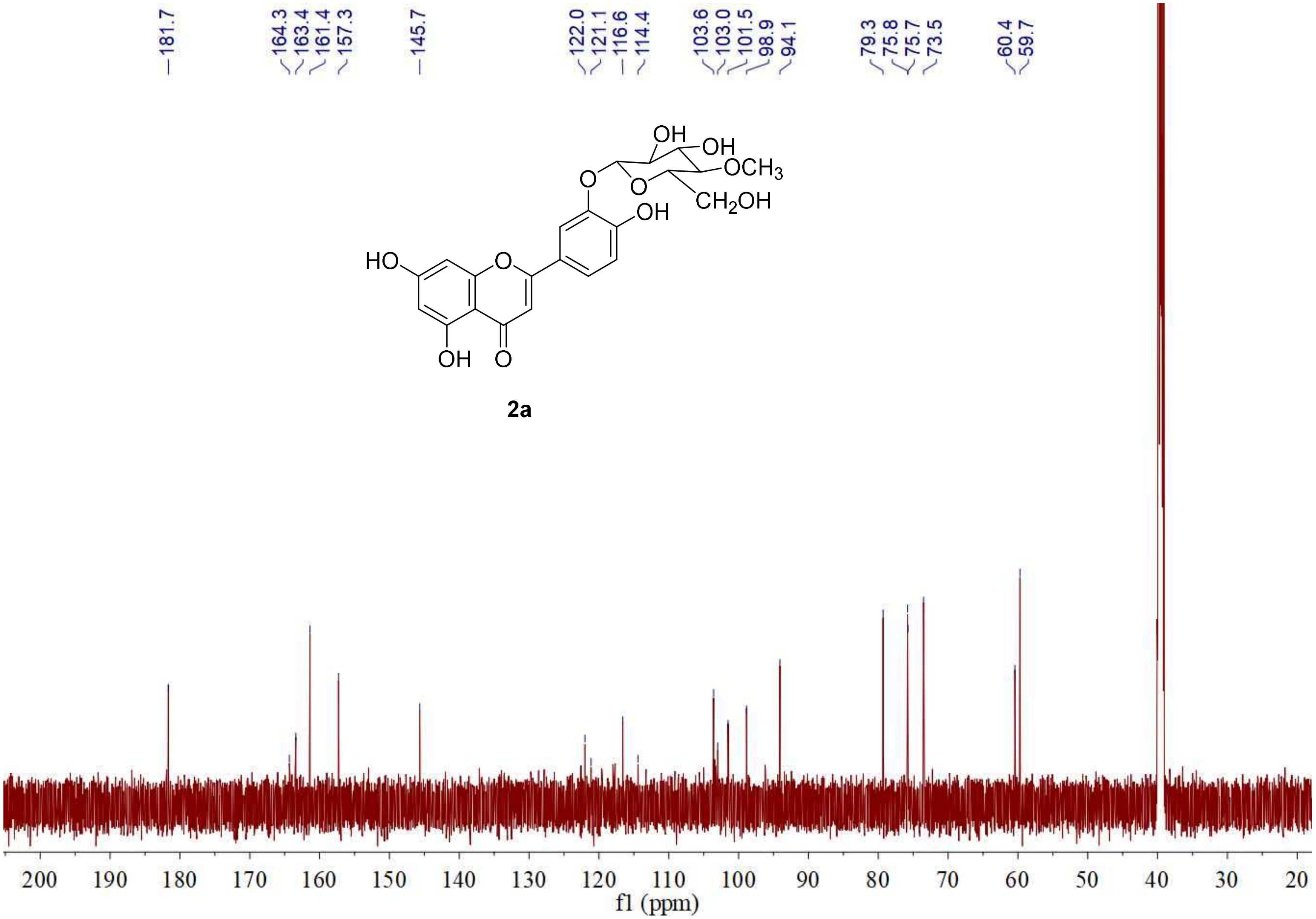

Figure S2.18 ^{13}C NMR spectrum of compound **2a** in DMSO-d_6

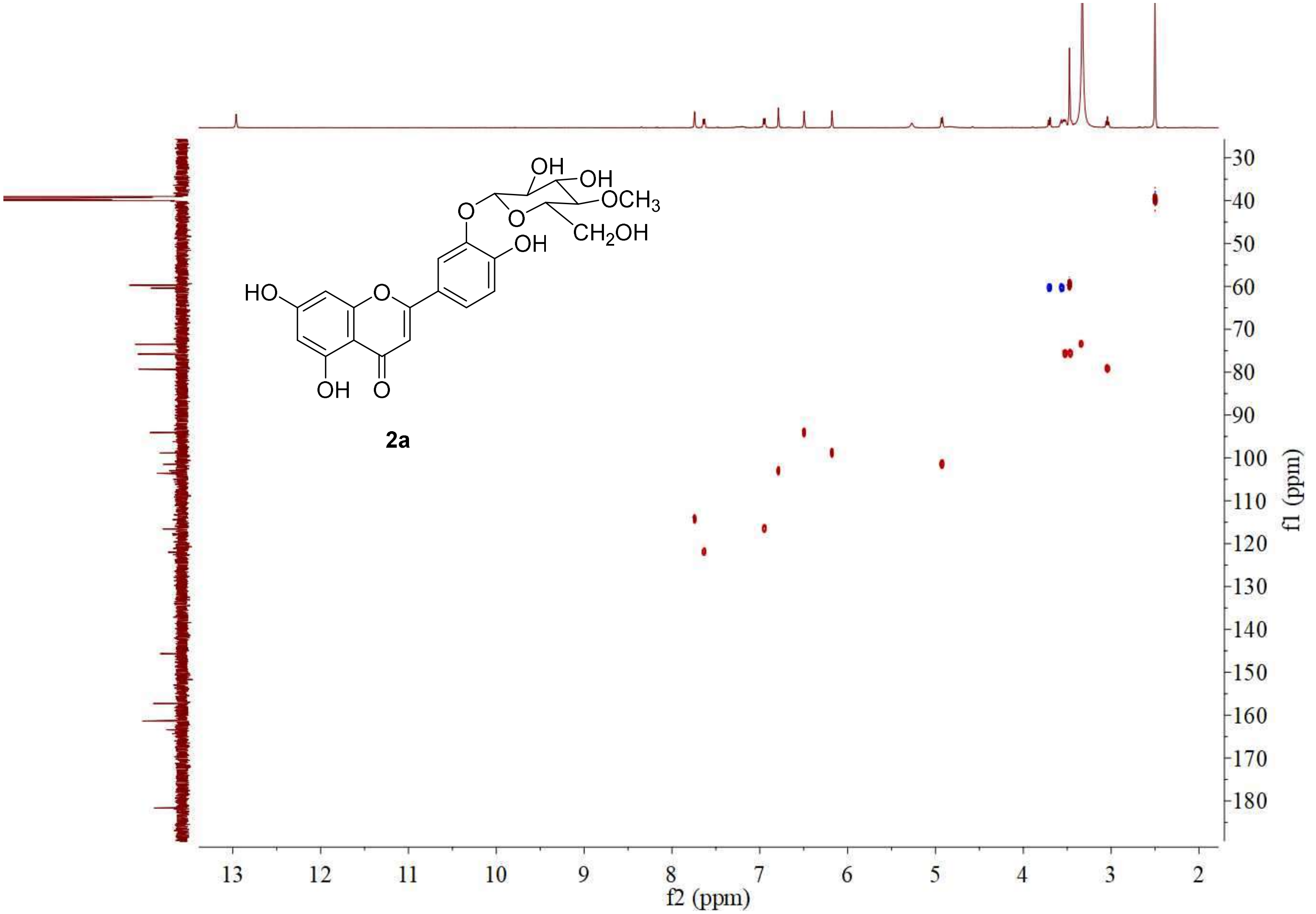

Figure S2.19 HSQC spectrum of compound **2a** in DMSO-d_6

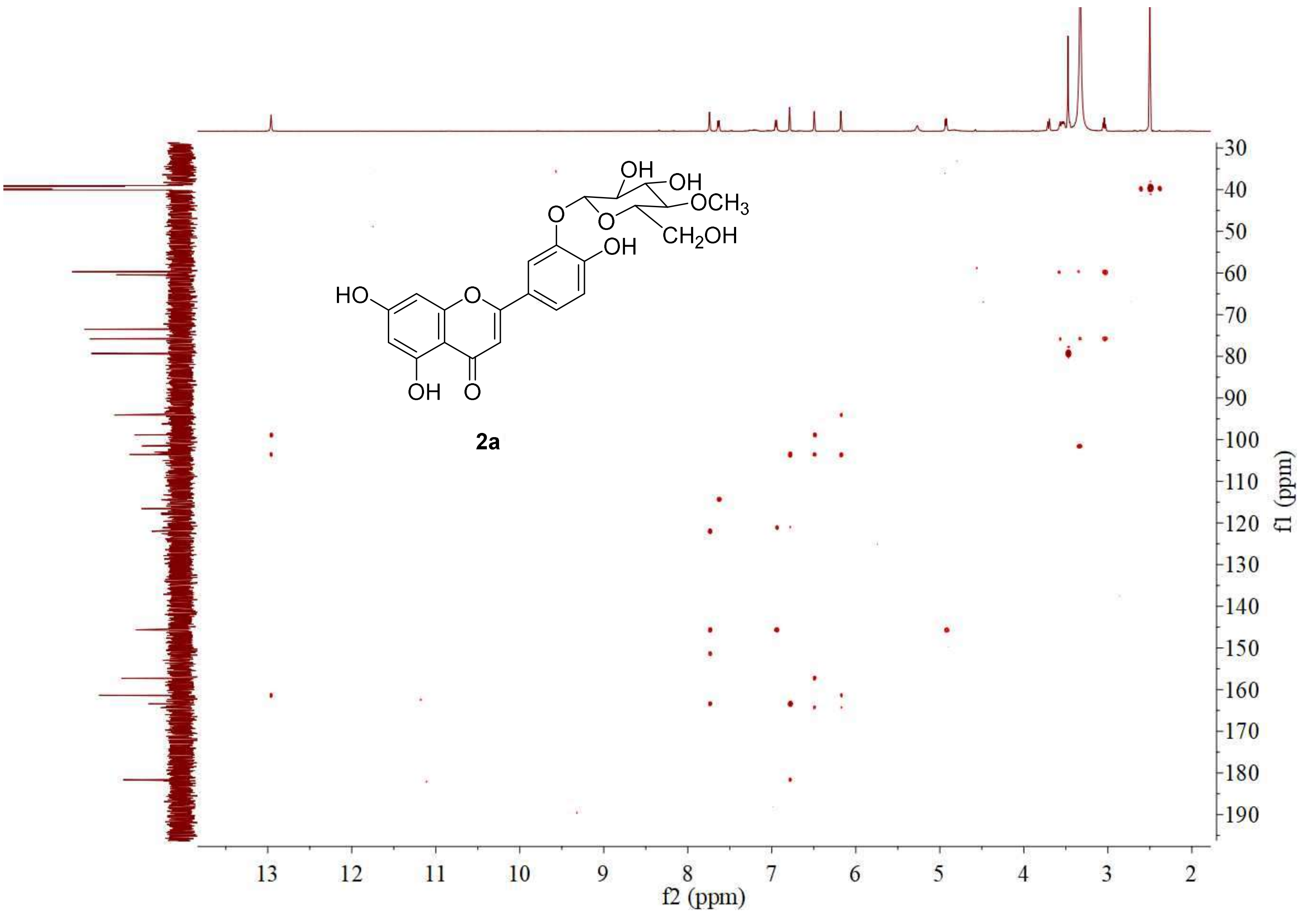

Figure S2.20 HMBC spectrum of compound **2a** in DMSO-d_6

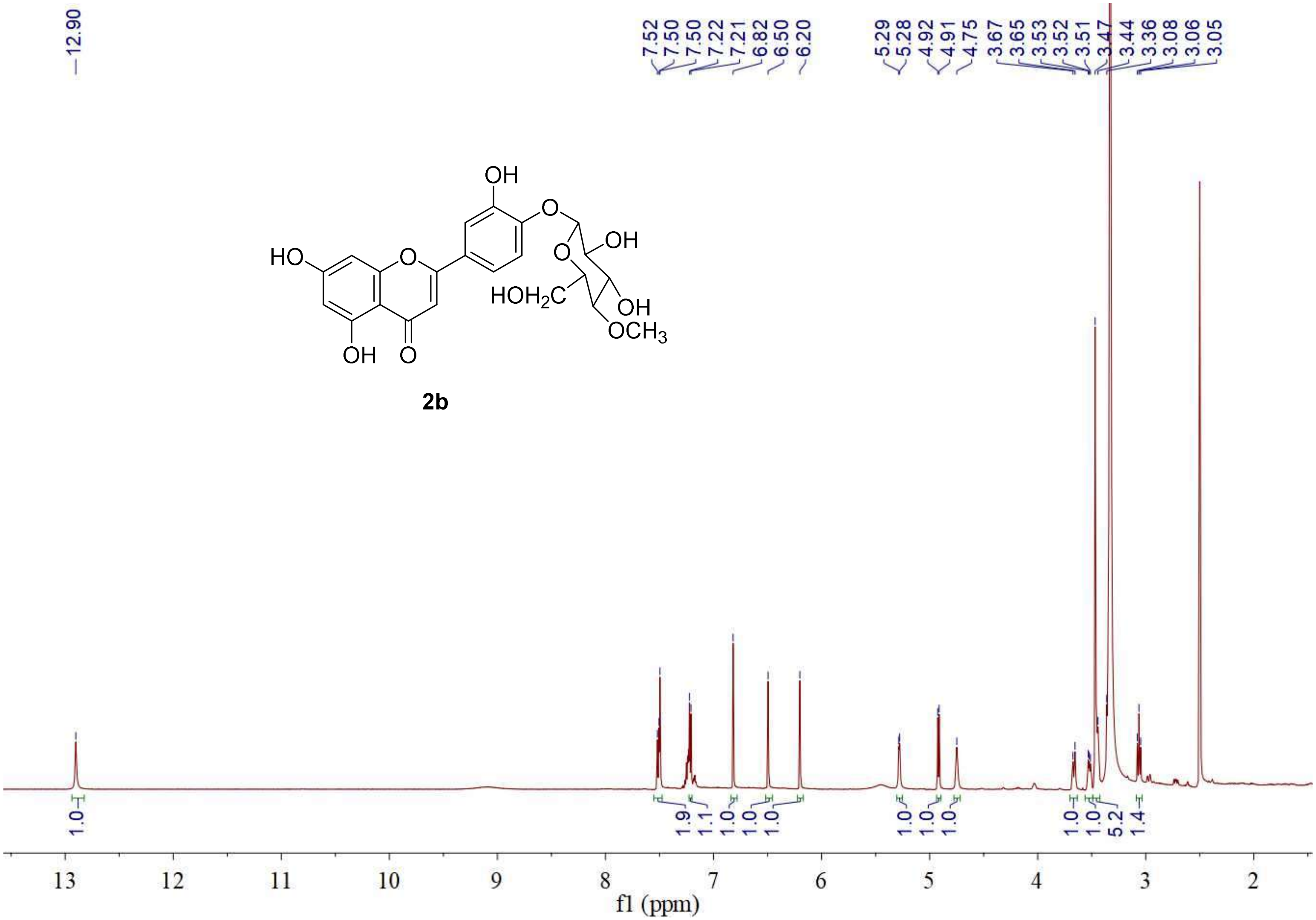

Figure S2.21 ^{1}H NMR spectrum of compound **2b** in DMSO-d_6

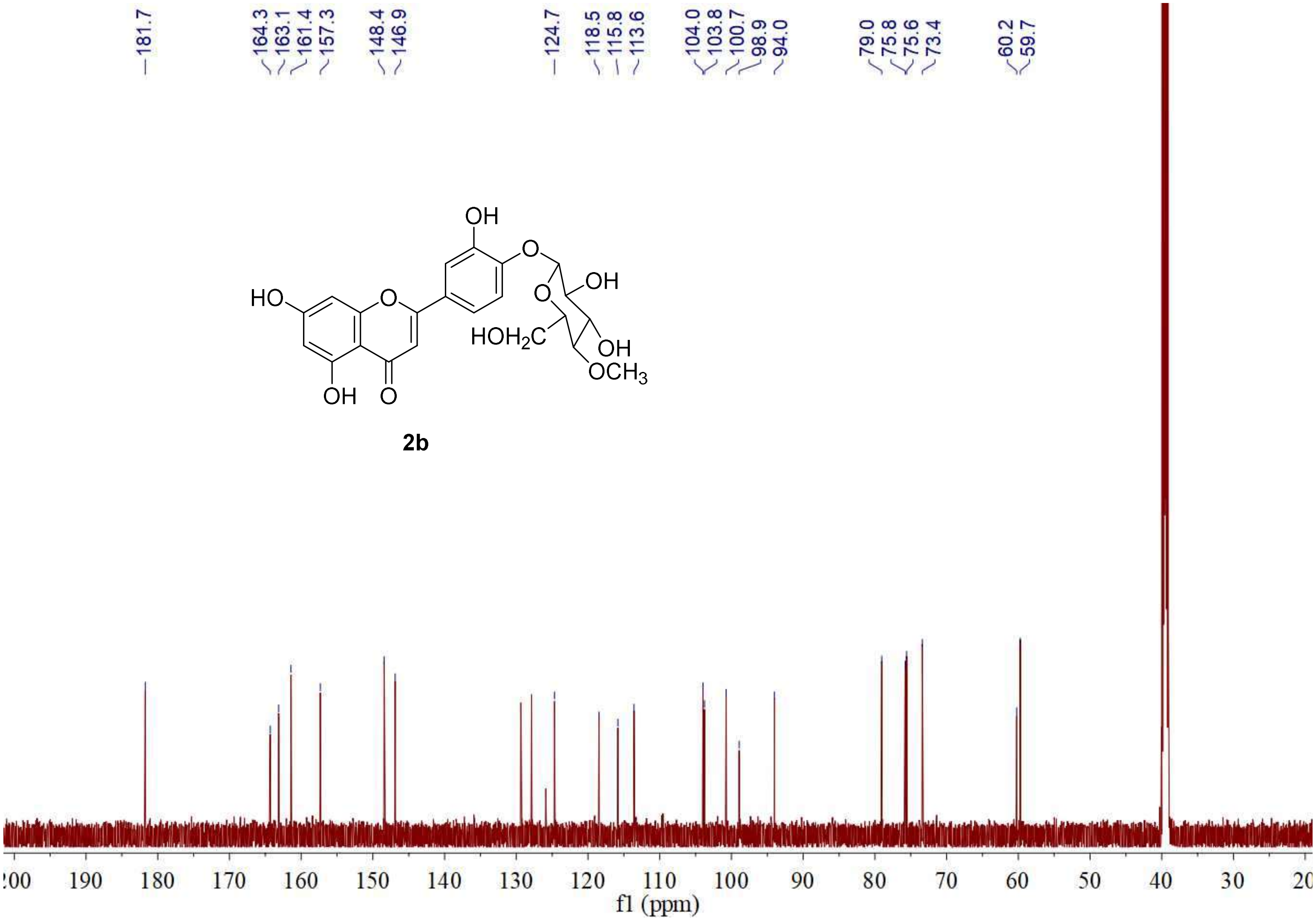

Figure S2.22 ^{13}C NMR spectrum of compound **2b** in DMSO-d_6

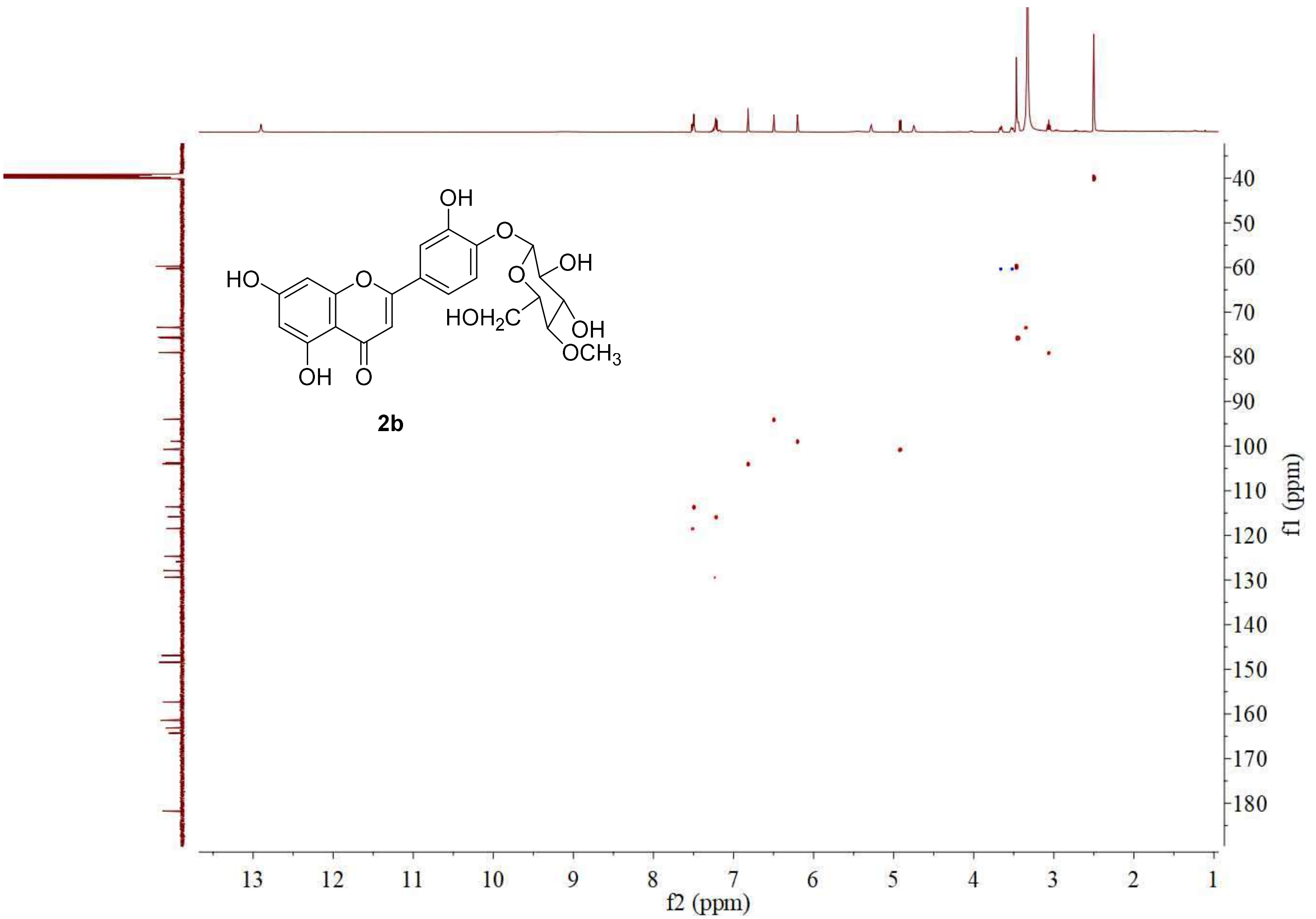

Figure S2.23 HSQC spectrum of compound **2b** in DMSO-d_6

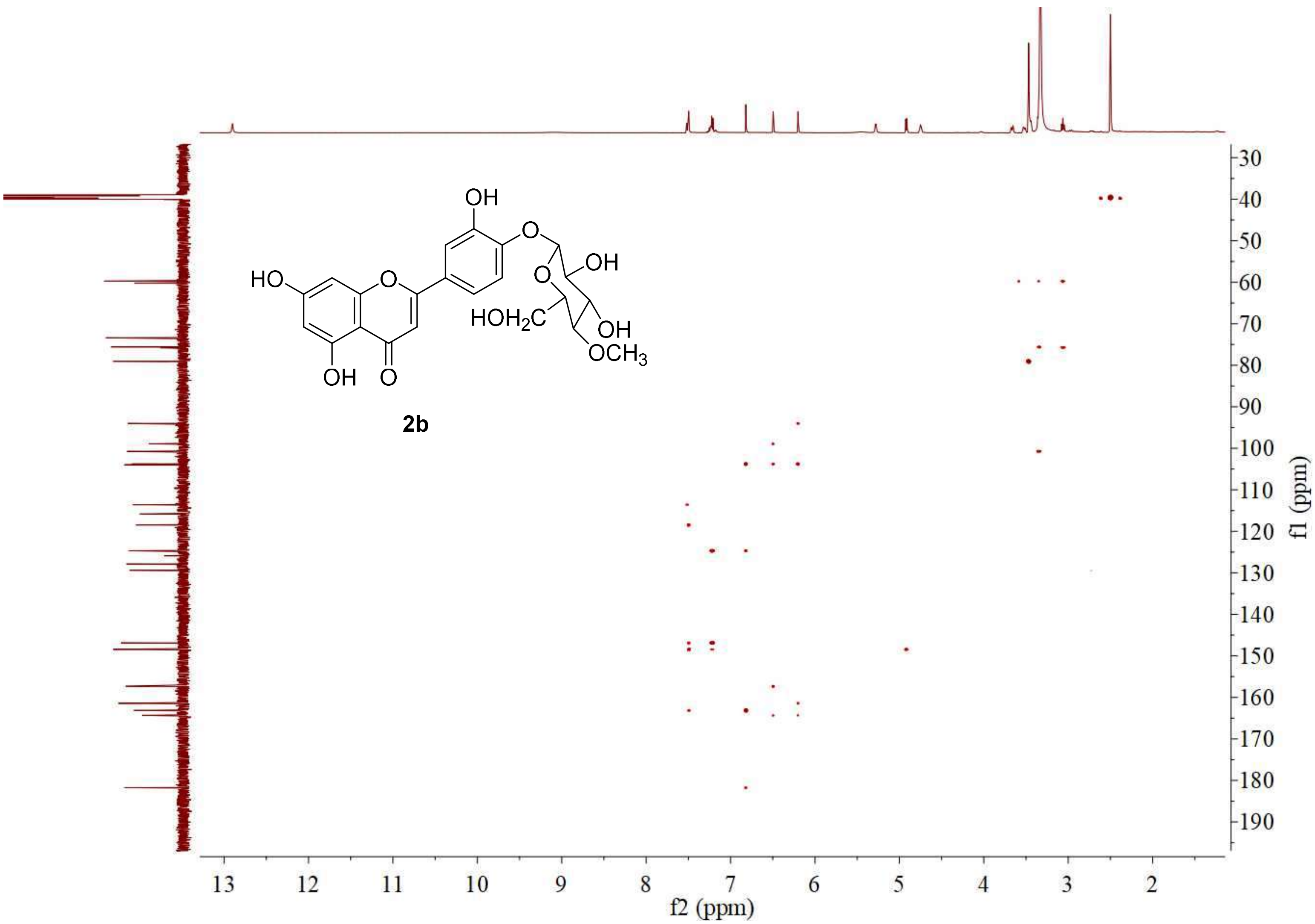

Figure S2.24 HMBC spectrum of compound **2b** in DMSO-d_6

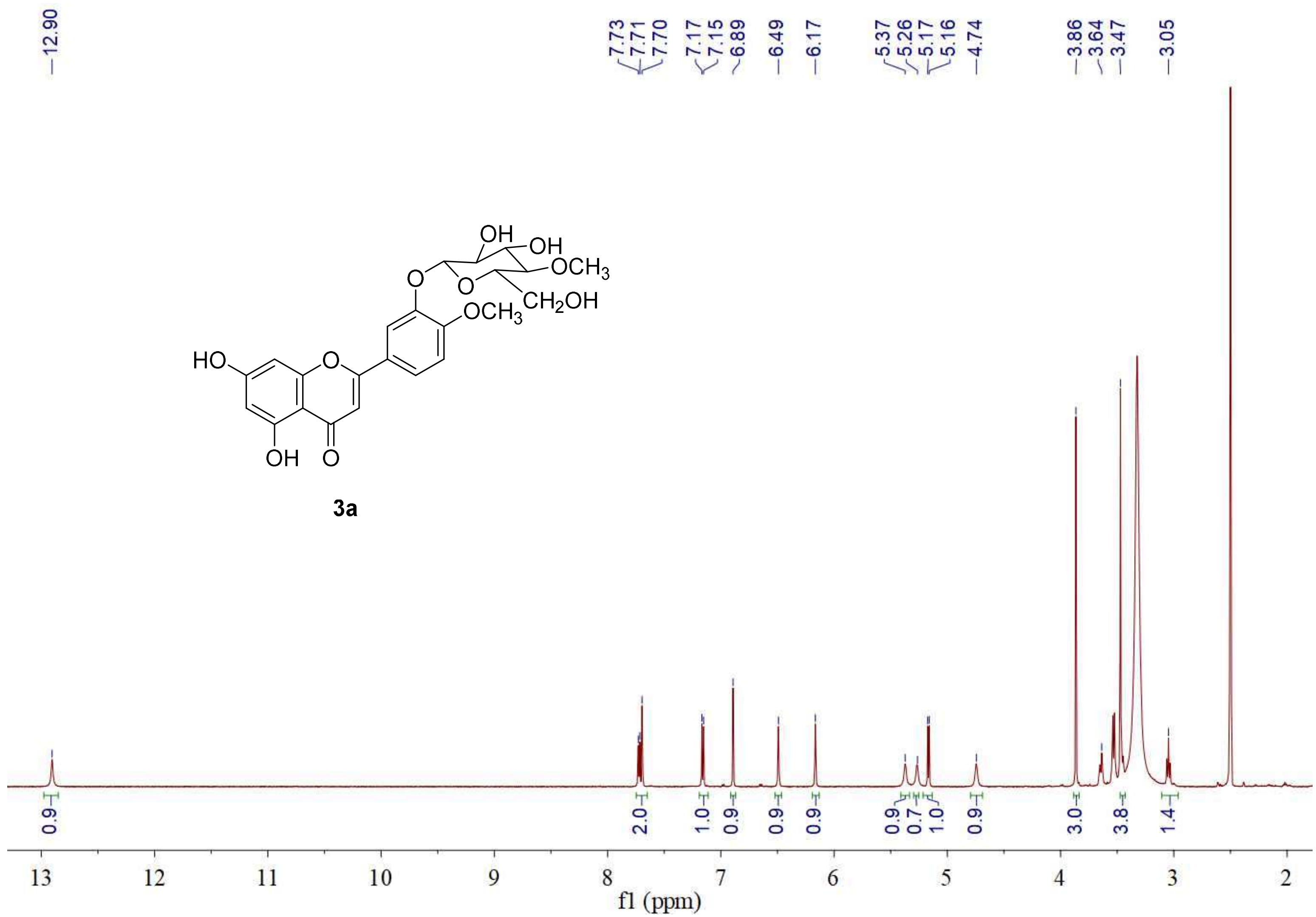

Figure S2.25 ^{1}H NMR spectrum of compound **3a** in DMSO-d_6

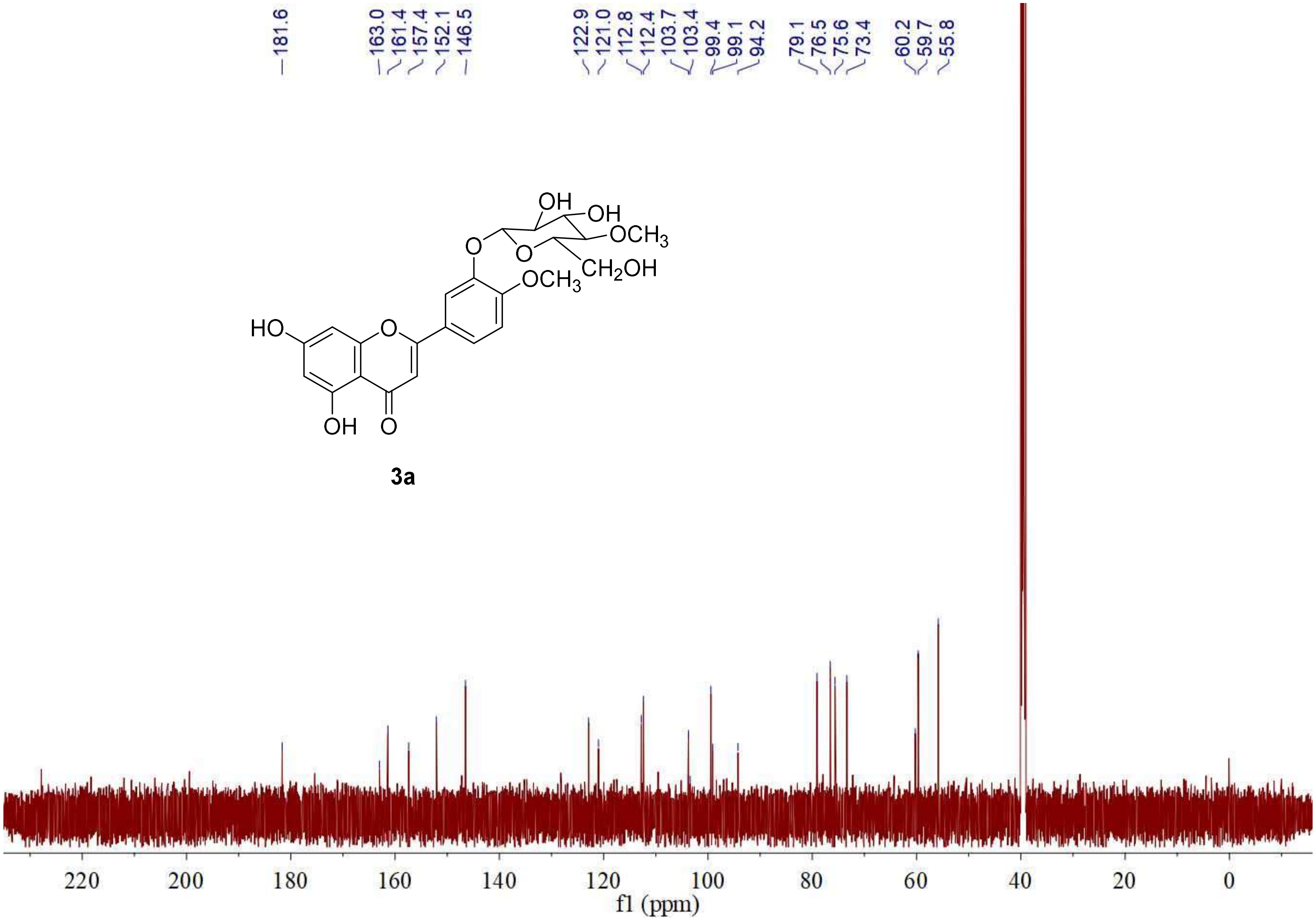

Figure S2.26 ^{13}C NMR spectrum of compound **3a** in DMSO-d_6

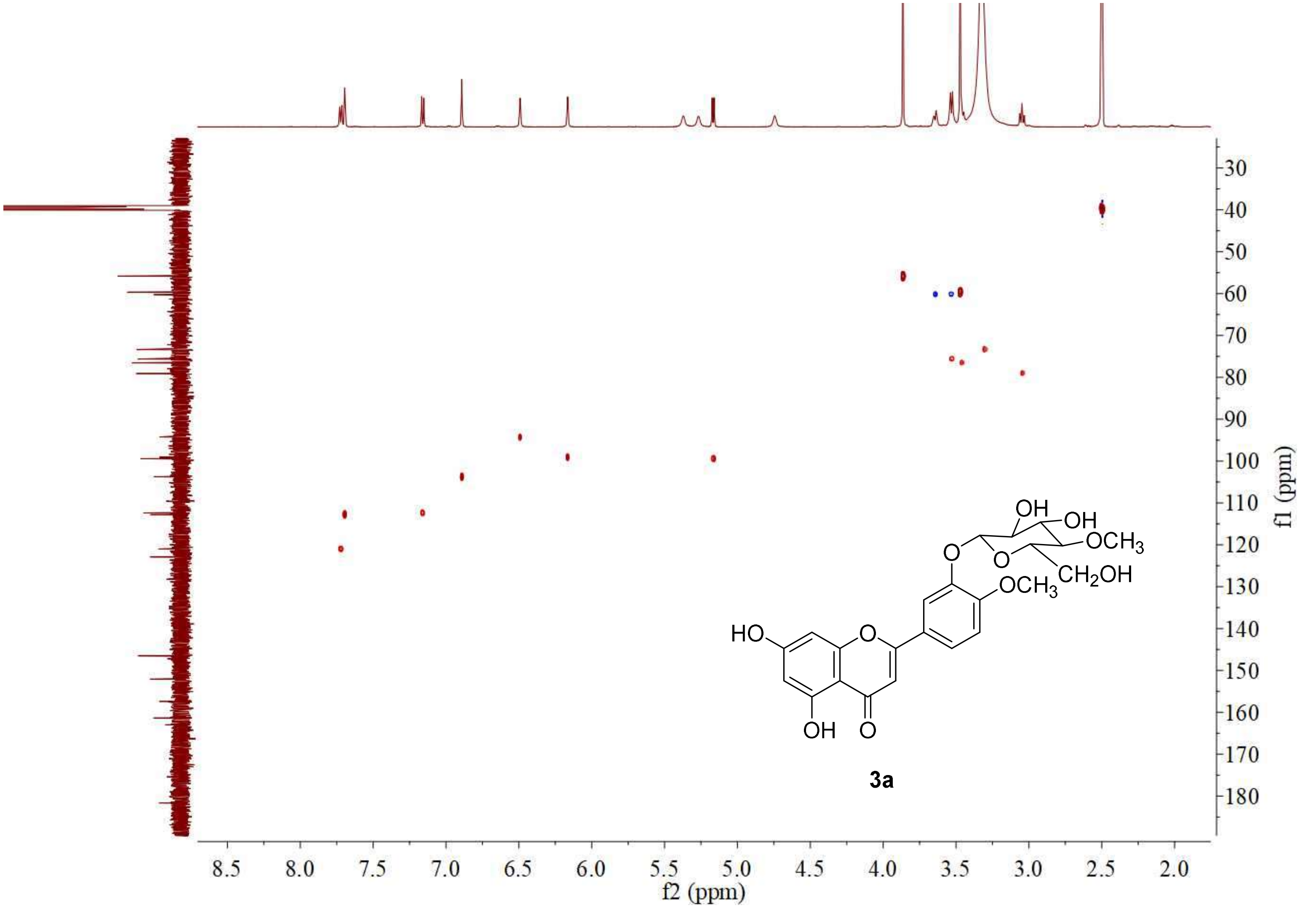

Figure S2.27 HSQC spectrum of compound **3a** in DMSO-d_6

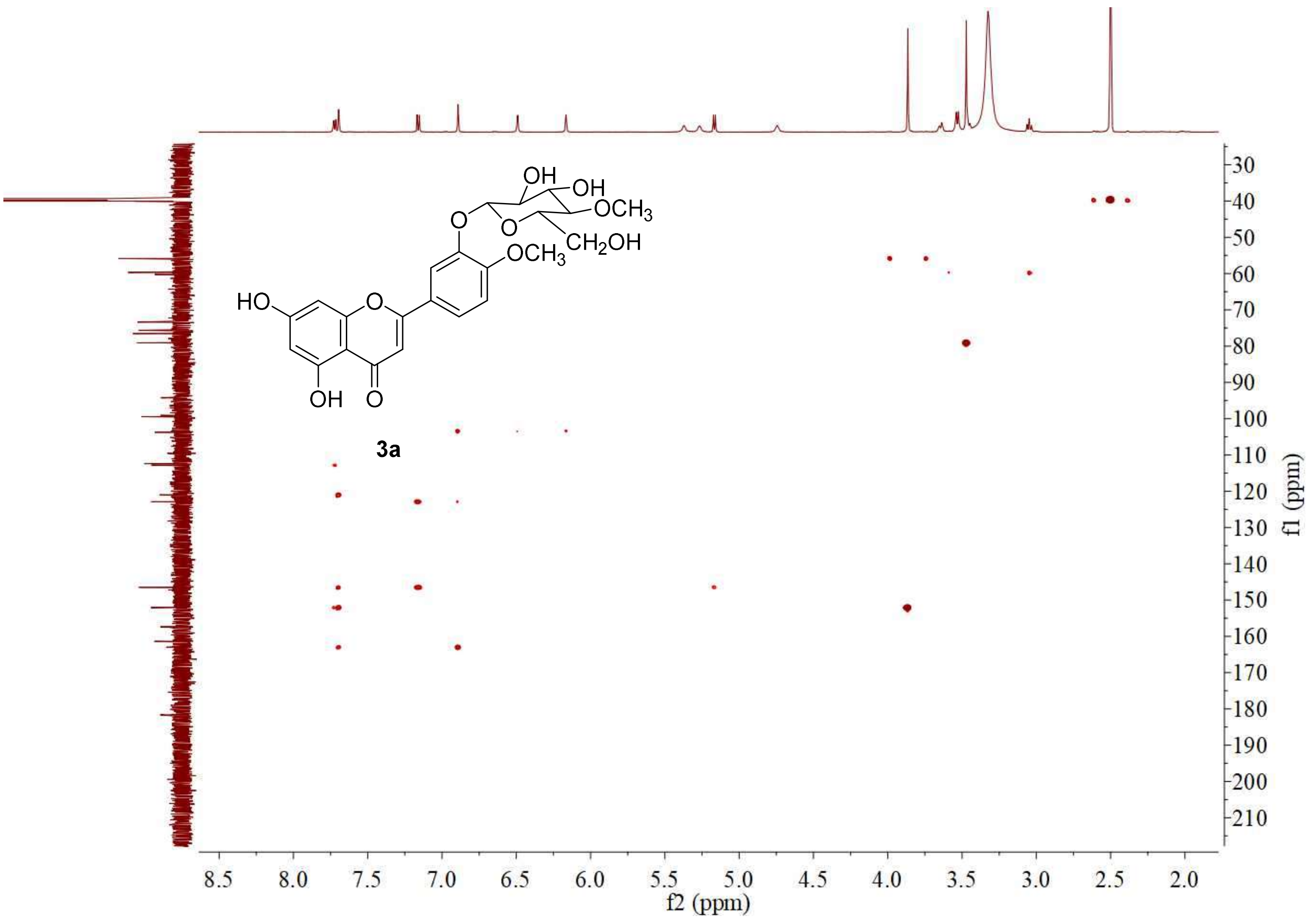

Figure S2.28 HMBC spectrum of compound **3a** in DMSO-d_6

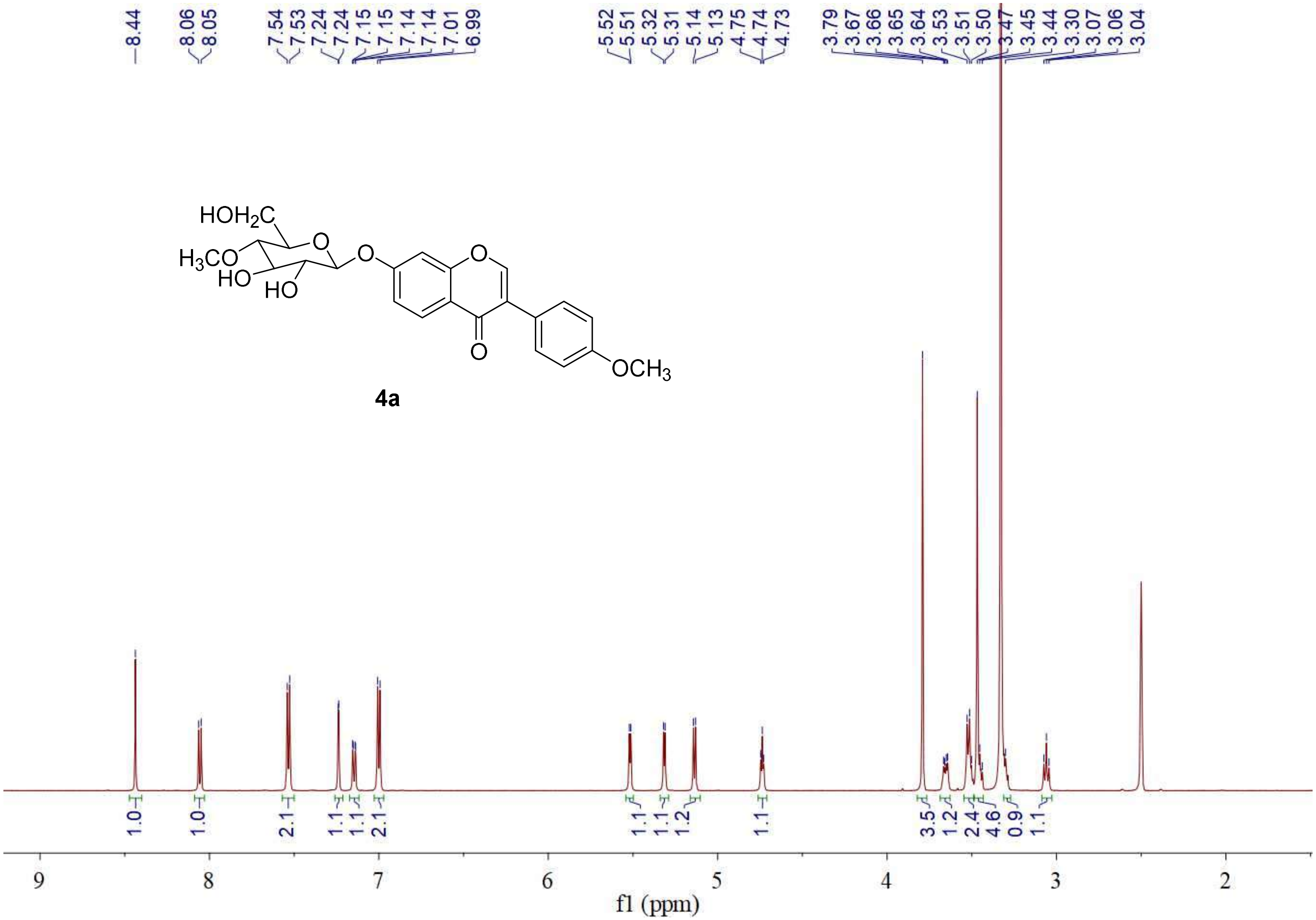

Figure S2.29 ^{1}H NMR spectrum of compound **4a** in DMSO-d_6

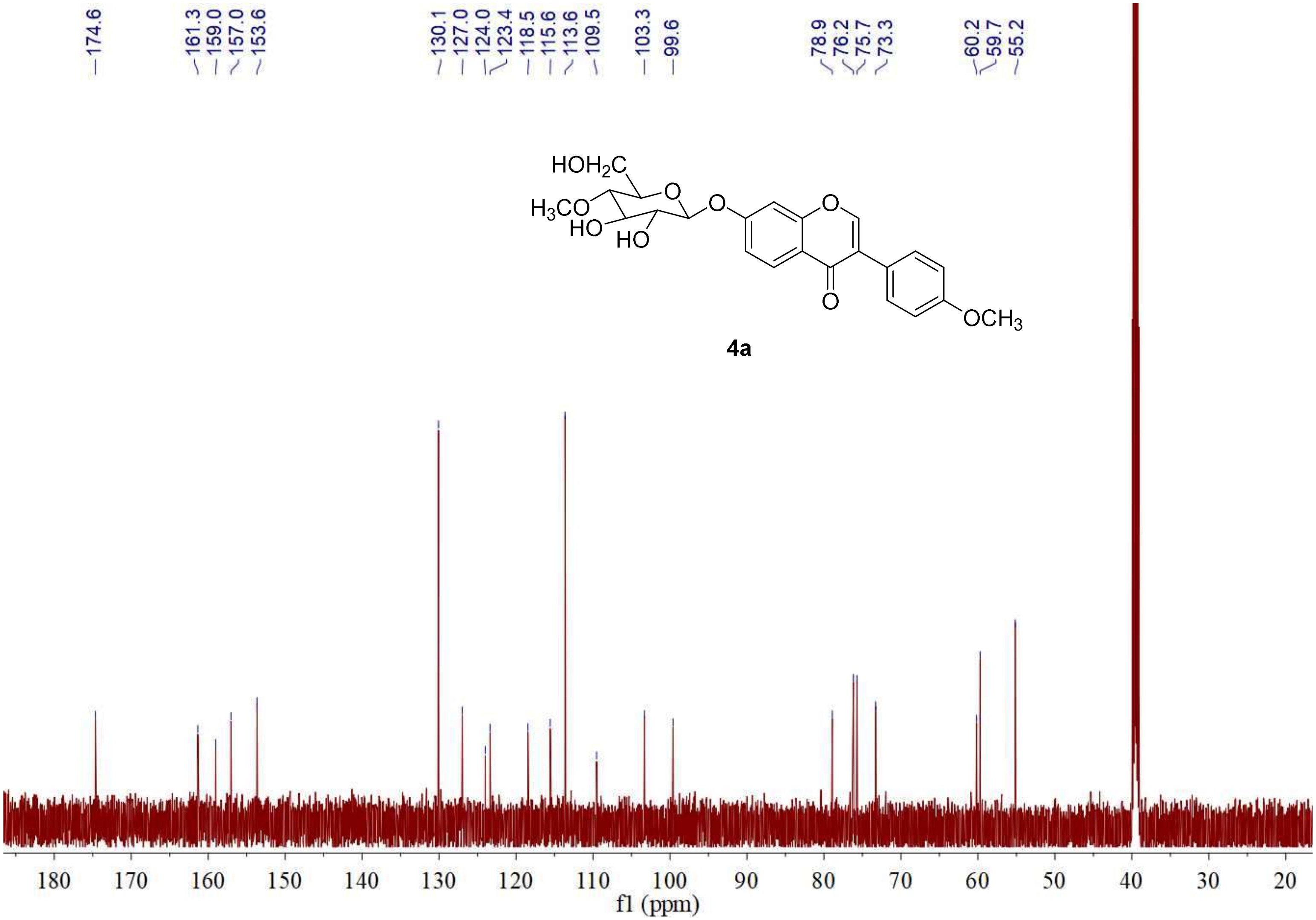

Figure S2.30 ^{13}C NMR spectrum of compound **4a** in DMSO-d_6

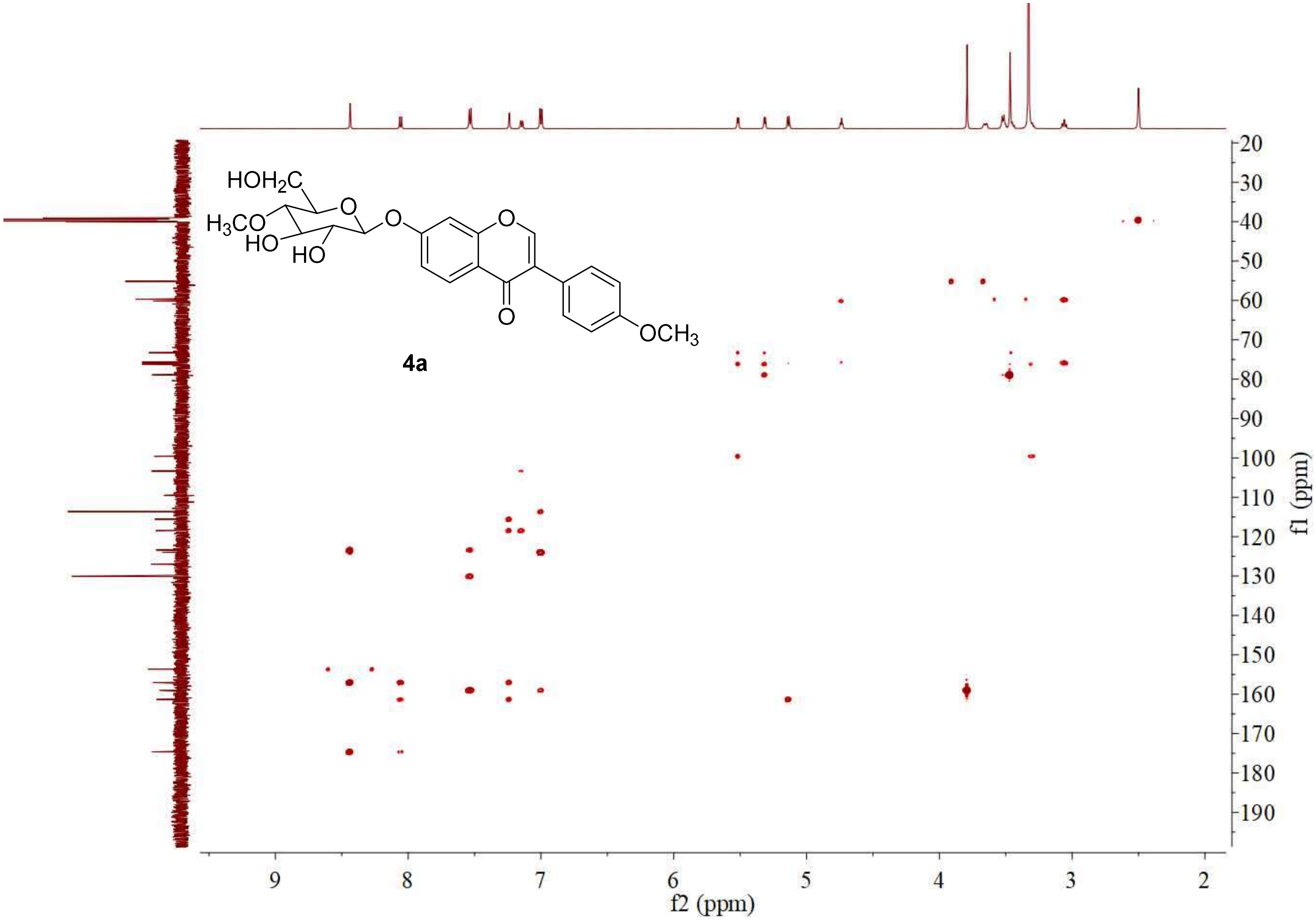

Figure S2.31 HMBC spectrum of compound **4a** in DMSO-d_6
